# Wearable Artificial Intelligence for Anxiety and Depression: Scoping Review

**DOI:** 10.2196/42672

**Published:** 2023-01-19

**Authors:** Alaa Abd-alrazaq, Rawan AlSaad, Sarah Aziz, Arfan Ahmed, Kerstin Denecke, Mowafa Househ, Faisal Farooq, Javaid Sheikh

**Affiliations:** 1 AI Center for Precision Health Weill Cornell Medicine-Qatar Doha Qatar; 2 Institute for Medical Informatics, Bern University of Applied Science Bern Switzerland; 3 Division of Information and Computing Technology, College of Science and Engineering, Hamad Bin Khalifa University, Qatar Foundation Doha Qatar; 4 Qatar Computing Research Institute, Hamad bin Khalifa University Doha Qatar

**Keywords:** wearable artificial intelligence, artificial intelligence, wearable devices, anxiety, depression, scoping review, mobile phone

## Abstract

**Background:**

Anxiety and depression are the most common mental disorders worldwide. Owing to the lack of psychiatrists around the world, the incorporation of artificial intelligence (AI) into wearable devices (wearable AI) has been exploited to provide mental health services.

**Objective:**

This review aimed to explore the features of wearable AI used for anxiety and depression to identify application areas and open research issues.

**Methods:**

We searched 8 electronic databases (MEDLINE, PsycINFO, Embase, CINAHL, IEEE Xplore, ACM Digital Library, Scopus, and Google Scholar) and included studies that met the inclusion criteria. Then, we checked the studies that cited the included studies and screened studies that were cited by the included studies. The study selection and data extraction were carried out by 2 reviewers independently. The extracted data were aggregated and summarized using narrative synthesis.

**Results:**

Of the 1203 studies identified, 69 (5.74%) were included in this review. Approximately, two-thirds of the studies used wearable AI for depression, whereas the remaining studies used it for anxiety. The most frequent application of wearable AI was in diagnosing anxiety and depression; however, none of the studies used it for treatment purposes. Most studies targeted individuals aged between 18 and 65 years. The most common wearable device used in the studies was Actiwatch AW4 (Cambridge Neurotechnology Ltd). Wrist-worn devices were the most common type of wearable device in the studies. The most commonly used category of data for model development was physical activity data, followed by sleep data and heart rate data. The most frequently used data set from open sources was Depresjon. The most commonly used algorithm was random forest, followed by support vector machine.

**Conclusions:**

Wearable AI can offer great promise in providing mental health services related to anxiety and depression. Wearable AI can be used by individuals for the prescreening assessment of anxiety and depression. Further reviews are needed to statistically synthesize the studies’ results related to the performance and effectiveness of wearable AI. Given its potential, technology companies should invest more in wearable AI for the treatment of anxiety and depression.

## Introduction

### Background

Anxiety and depression are among the common mental illnesses with a high global prevalence. It was reported that as of 2020, a total of 19% of people worldwide were living with depression or anxiety, which prevented them from doing their daily activities as they normally would have for ≥2 weeks [1]. In addition to having a significant economic impact on society [2], anxiety and depression affect people in terms of years lost because of illness. The statistics are astounding; depression is the world’s leading cause of disability within the youth population [3-5]. As per a study among US adults, at 18 years of age, adults with depression had 28 more years of quality-adjusted life expectancy than adults without depression, resulting in a 28.9-year quality-adjusted life expectancy loss owing to depression [6]. Depression is also a significant risk factor when it comes to suicide [7]. Given the abovementioned statistics and the fact that there are only approximately 9 psychiatrists per 100,000 people in high-income countries [8] and 0.1 per 1 million people in low-income countries [9], the situation is challenging to say the least. Current approaches for the assessment of anxiety and depression disorders are primarily based on the clinical observation of patients’ mental states, clinical history, and self-report questionnaires, such as the Generalized Anxiety Disorder-7 for anxiety and Patient Health Questionnaire-9 for depression. However, these methods are subjective, time consuming, and challenging to repeat. As a result, contemporary psychiatric assessments can be inaccurate and ineffective at assessing anxiety and depression symptoms in a reliable and personalized manner. Therefore, there is a significant need to develop automatic techniques to address the limitations of the current psychiatric approaches for assessing anxiety and depression disorders and overcome the shortages and uneven distribution of mental health professionals.

Recently, there have been rapid ongoing developments in artificial intelligence (AI) technology and wearable technology for health care and clinical use, offering numerous advantages for individualizing diagnoses and the treatment management of psychiatric disorders, including anxiety and depression [10-12]. Wearable technology includes electronic devices that users can wear near the body (eg, smartwatches, smart glasses, and smart bracelets), on the body (eg, electrocardiogram electrodes), and in the body (eg, implantable smart patches) and electronic textiles (eg, smart clothes). Wearable devices are designed to provide a constant stream of health care data for disease diagnosis and treatment. This is achieved by continuously recording physiological parameters such as temperature; blood pressure; blood oxygen; respiratory rate; physical movement; and the electrical activity of the heart, brain, and skin. Symptoms of anxiety and depression can be assessed by many parameters collected in real time by wearable devices for the diagnosis and monitoring of patients with anxiety and depression.

However, the dramatically accelerating pace of the development and adoption of wearables coupled with a shortage of skilled caregivers has led to an evolving need for automatic, efficient, and real-time approaches to analyze the large volumes of data collected by wearable sensors. This has motivated the integration of AI methods into wearable devices, introducing the “Wearable AI” technology. Wearable AI refers to intelligent electronic devices that are designed to be worn on the user’s body and possess intelligent operations. Wearable devices typically deal with monitoring and analyzing patients’ health data. However, when paired with AI, wearable devices introduce fundamental developments in the diagnosis and treatment of anxiety and depression. It has the potential to provide an early and accurate diagnosis of anxiety and depression, facilitate more individualized treatment for patients with anxiety and depression, and assist in developing preventive measures for groups at the risk of anxiety and depression.

### Research Problem and Aim

Several studies were published on wearable devices combined with AI for the treatment of anxiety and depression. Several reviews were conducted to summarize previous studies; however, they had the following limitations. First, they focused on wearable devices rather than wearable devices paired with AI [10-15]. Second, they did not describe in detail the features of the used wearable devices and AI models [10-15]. Third, they targeted only certain age groups, such as children and adolescents [10,12]. Fourth, they focused on wearable devices for either anxiety [11,14] or depression [12,13,15] rather than both anxiety and depression. Fifth, they did not search relevant databases, such as MEDLINE [14], PsycINFO [10,13,15], IEEE Xplore [10-14], and ACM Digital Library [10-15]. Finally, they focused on wearable devices used for only diagnostic purposes using only electrocardiogram data [11] or electroencephalogram data [15]. Therefore, the need for a review that focuses on AI-paired wearable devices for anxiety and depression has never been higher. The quality of this review should as high as that of a previous review conducted on AI-paired wearable devices for diabetes [16]. The current review aims to explore the features of wearable AI used for anxiety and depression to both help customers make educated selections and help the research community advance in this field by identifying gaps and examining future prospects.

## Methods

### Overview

To achieve the objective of the study, we conducted a scoping review consistent with PRISMA-ScR (Preferred Reporting Items for Systematic Reviews and Meta-Analyses Extension for Scoping Reviews) [17]. PRISMA-ScR checklist for this review is presented in Multimedia Appendix 1 [17]. The methods used in this review are detailed in the following subsections.

### Search Strategy

To identify relevant studies, we searched 8 electronic databases on May 30, 2022: MEDLINE (via Ovid), PsycINFO (via Ovid), Embase (via Ovid), CINAHL (via EBSCO), IEEE Xplore, ACM Digital Library, Scopus, and Google Scholar. We set up an automatic biweekly search for 24 weeks (ending on September 30, 2022). Given that Google Scholar retrieved a massive number of hits and ordered them based on their relevance, only the first 100 hits (ie,10 pages) were checked in this review. To identify additional studies, we checked the reference lists of the included studies (ie, backward reference list checking) and screened studies that cited the included studies (ie, forward reference list checking).

To develop the search query, 3 experts in digital mental health were consulted, and relevant previous reviews were checked. The search query was composed of 3 groups of terms: terms related to AI (eg, artificial intelligence, machine learning, and deep learning), terms related to wearable devices (eg, wearable OR smart watch OR smartwatch), and terms related to anxiety and depression (eg, anxiety OR anxious OR depression). Multimedia Appendix 2 presents the detailed search query used for searching each database.

### Study Eligibility Criteria

This review included studies that focused on developing AI algorithms for anxiety and depression using data collected by wearable devices. Specifically, we focused on all AI algorithms used for any purpose related to anxiety and depression (eg, diagnosis, monitoring, screening, therapy, prediction, and prevention). The wearable devices that were used for collecting data had to be noninvasive on-body wearables, such as smartwatches, smart glasses, smart clothing, smart bracelets, and smart tattoos. By contrast, we excluded studies that used data collected by the following devices: nonwearable devices, handheld devices (eg, mobile phones), near-body wearable devices, in-body wearable devices (eg, implants), wearable devices connected to nonwearable devices using wires, and wearable devices that can be placed on users only by an expert (eg, wearable devices composed of many electrodes that need to be placed in very specific points of the body). Studies that used data collected via any method (eg, nonwearable devices, questionnaires, and interviews) in addition to via wearable devices were considered in this review. We excluded studies that showed only a theoretical framework of AI-based wearable devices for anxiety and depression. We included journal articles, conference papers, and dissertations that were published in the English language since 2015. We excluded reviews, preprints, conference abstracts, posters, protocols, editorials, and commentaries. No restrictions were enforced regarding the measured outcomes, setting, or country of publication.

### Study Selection

We followed 3 steps in the study selection process. In the first step, we used EndNote X9 (Clarivate Plc) to remove duplicates from all the retrieved studies. In the second step, we checked the titles and abstracts of the remaining publications. Finally, we screened the entire texts of the studies selected in the previous step. Two reviewers independently performed the study selection process. Disagreements between them in the second and third steps were resolved through discussion. Cohen κ was calculated to measure the interrater agreement [18], and it was 0.85 for “title and abstract” screening and 0.92 for full-text reading.

### Data Extraction

Two reviewers used Excel (Microsoft Corp) to independently extract data on study metadata, wearable devices, and AI techniques. Any disagreements between the reviewers were resolved through discussion. The data extraction form used in this review was piloted using 5 studies and is shown in Multimedia Appendix 3.

### Data Synthesis

Data extracted from the included studies were synthesized using the narrative approach, wherein data were summarized and described using texts, tables, and figures. More specifically, we began by describing the metadata of the included studies (eg, year of publication and country of publication). Then, we presented the features of the wearable devices used in the included studies (eg, their status, type, placement, and operating system). Finally, we summarized the characteristics of the AI techniques used (eg, AI algorithms used, their aim, data set size, and data input type). We used Microsoft Excel to manage data synthesis.

## Results

### Search Results

**Figure 1:**
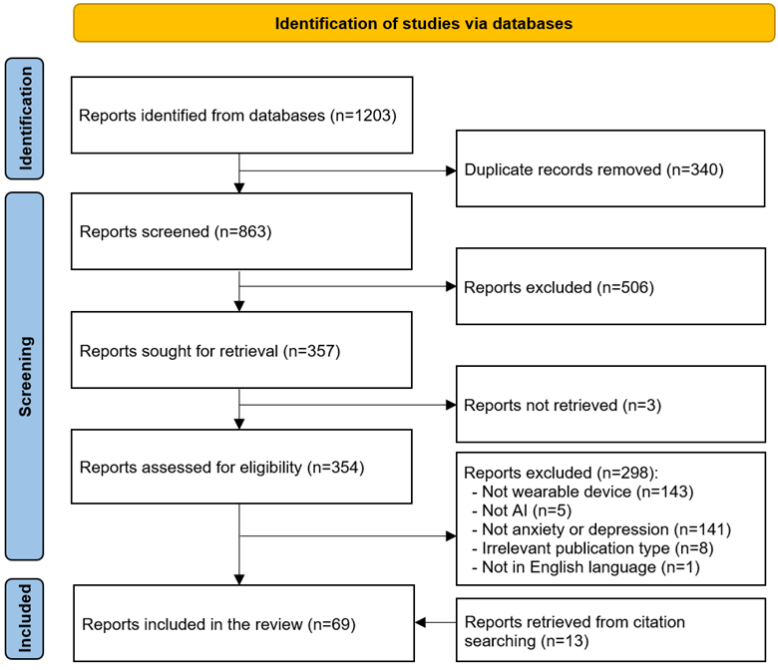
Flowchart of the study selection process. AI: artificial intelligence.

As shown in Figure 1, searching all preidentified databases retrieved 1203 records. Of these 1203 records, 340 (28.26%) duplicates were detected and removed using a reference management software (EndNote X9). Screening the titles and abstracts of the remaining 71.74% (863/1203) of records resulted in the exclusion of 58.6% (506/1203) of records. Of the remaining 41.4% (357/1203) of records, we could not find the full text of 0.8% (3/1203) of records. Reading the full text of the remaining 99.2% (354/357) of records led to the exclusion of 84.2% (298/357) of records for several reasons, which are shown in Figure 1. We identified 13 additional records relevant to this review by backward and forward reference list checking. In total, 69 records were included in this review [19-87].

### Characteristics of the Included Studies

The included studies were published between 2015 and 2022 (Table 1). The year in which the largest number of included studies was published was 2021 (17/69, 25%), followed by 2019 (16/69, 23%) and then 2020 (15/69, 22%). The studies were conducted in 21 countries (Table 1). More than a quarter (21/69, 30%) of the studies were published in the United States. The included studies were peer-reviewed journal articles (49/69, 71%), conference proceedings (18/69, 26%), and theses (2/69, 3%).

The number of participants in the included studies ranged from 8 to 4036, with an average of 186.7 (SD 522.2; Table 1). The mean age of the participants was reported in 72% (50/69) of studies and ranged between 5.2 and 78 years, with an average of 36.4 (SD 15.4) years. Only 9% (6/69) of the included studies targeted children (aged <18 years), and 4% (3/69) of studies focused on only older adults (aged ≥65 years). The percentage of female participants was reported in 54 studies and varied between 2.4% and 100%, with an average of 59.8% (SD 15.3%). More than one-third (26/69, 38%) of the studies recruited individuals with any health condition, and approximately 30% (21/69) of the studies included both patients with depression and healthy individuals. Multimedia Appendix 4 [19-87] shows the characteristics of each included study.

**Table 1:** Characteristics of the included studies (N=69).

Features		Values	References
**Year of publication, n (%)**			
	2022	10 (13)	[27,30,38,48,52,59,63,64,69,81]
	2021	17 (25)	[19-21,23,25,28,41,45,49,54,61,62,68,73,74,77,78]
	2020	15 (22)	[22,29,31,33,40,43,44,53,57,60,66,70,71,76,79]
	2019	16 (23)	[26,32,34,42,46,47,51,56,65,67,72,75,80,84-86]
	2018	5 (7)	[35,36,50,55,83]
	2017	4 (6)	[24,37,39,58]
	2016	1 (1)	[87]
	2015	1 (1)	[82]
**Type of publication, n (%)**			
	Journal article	49 (71)	[19,21,23,25-30,34,38-46,48-54,56-61,64-66,69-71,73-75,77-79,81,82,84,86,87]
	Conference paper	18 (26)	[20,22,24,31-33,35-37,55,62,63,67,68,72,80,83,85]
	Thesis	2 (3)	[47,76]
**Country of publication, n (%)**			
	United States	21 (30)	[24,25,30,31,37,41,42,50,54-56,59,61,66,74,76,77,80,83-85]
	Mexico	7 (10)	[34,58,65,69-71,86]
	Norway	6 (9)	[20,32,35,36,43,47]
	United Kingdom	5 (7)	[29,38,48,72,78]
	South Korea	5 (7)	[26-28,46,60]
	Japan	4 (6)	[33,63,67,79]
	Pakistan	3 (4)	[21,22,45]
	China	3 (4)	[23,39,44]
	India	2 (3)	[51,53]
	Taiwan	2 (3)	[62,81]
	Others	11 (16)	[19,40,49,52,57,64,68,73,75,82,87]
**Number of participants**			
	Mean (SD; range)	186.9 (522.2; 8-4036)	[19-87]
	1-100, n (%)	53 (77)	[19-22,24,26,27,30-40,42-49,53,55,56,58-68,70,71,75-79,81-83,85-87]
	101-500, n (%)	11 (16)	[23,25,41,50,51,54,57,69,73,80,84]
	>500, n (%)	5 (7)	[28,29,52,72,74]
**Age of the participants (years)**			
	Mean (SD; range)	36.4 (15.44; 5.2-78)	[19-21,26-30,32,34-38,41-43,46-48,51,52,54-59,61-71,73-80,83,85,86]
	<18, n (%)	5 (7)	[54-56,59,76]
	18-40, n (%)	17 (25)	[21,26,29,37,51,52,58,66,67,73-75,77,78,80,83,85]
	41-65, n (%)	25 (36)	[19,20,27,30,32,34-36,38,41-43,47,48,57,62-65,68-71,79,86]
	>65, n (%)	3 (4)	[27,46,61]
Sex (female; %), mean (SD; range)		59.4 (15.64; 2.4-100)	[19-22,26-32,34-38,41-48,50,52,53,55-59,61-71,73,75-81,83,85,86]
**Participant health conditions^a^, n (%)**			
	Depression	32 (46)	[19,20,23,24,26,30,32,34-38,42,43,46-48,50,57,59,60,62,65-71,77,79,86]
	Healthy	27 (39)	[19,20,32,34-36,42,43,47,48,50,54-57,60,62,65,67-71,75,76,79,86]
	Any health condition	26 (38)	[21,22,25,27-29,31,33,39,41,44,45,52,53,58,61,63,64,72-74,80,83-85,87]
	Internalizing disorders	4 (6)	[54-56,76]
	Bipolar	3 (4)	[26,49,82]
	Others	6 (9)	[40,51,69,75,78,81]

^a^Numbers do not add up, as participants in many studies had >1 health condition.

### Features of Wearable Devices

The included studies focused on wearable devices for depression (44/69, 64%), anxiety (17/69, 25%), or both (8/69, 12%). Approximately, 90% (62/69) of the included studies used commercial wearable devices (Table 2). The included studies used 41 different wearable devices. All studies, except for 7, used only 1 wearable device. The most common wearable device used in the included studies was Actiwatch AW4 (Cambridge Neurotechnology Ltd; 17/69, 25%), followed by Fitbit (Fitbit Inc) series (eg, Fitbit Charge, Fitbit Flex, and Fitbit Altra; 13/69, 19%) and Empatica (Empatica Inc) series (eg, E3 and E4; 7/69, 10%). The commercial wearable devices were manufactured by 25 different companies, the most common companies of which was Cambridge Neurotechnology (17/69, 25%), followed by Fitbit Inc (13/69, 19%) and Empatica Inc (7/69, 10%). Multimedia Appendix 5 [19-87] shows the features of the wearable devices in each included study.

The wearable devices in the included studies were available in 7 forms, but the most common form was smart bands (50/69, 72%), followed by smartwatches (16/69, 23%; Table 2). The wearable devices in the included studies were worn on 11 different parts of the body, but wrist-worn devices were the most common (57/69, 83%) in the included studies. The compatibility of the wearable devices with the operating systems of other devices was identified in 61 studies. The wearable devices were compatible with only 1 operating system in 41% (25/61) of the studies and >1 operating system in 59% (36/61) of the studies. The most common operating systems compatible with the wearable devices in the included studies was Windows (Microsoft Corp; 52/61, 85.2%), followed by iOS (Apple Inc; 36/61, 59%) and Android (35/61, 57%).

Only 21 studies (30%) used a gateway between the wearable device and the main host device (Table 2). In 62% (13/21) of studies, the gateways were PCs, smartphones, and tablets. The included studies used 4 types of host devices (ie, end gate devices that store data collected by wearable devices). More than one host device was used in 20% (14/69) of studies. The most common host device in the included studies was computer (46/69, 67%), followed by database server (30/69, 43%). Data were transferred from the wearable device to the host device through 6 different modes. In approximately 46% (32/69) of the studies, >1 mode of data transfer was used. The most common mode was Bluetooth (41/69, 59%), followed by docking stations (27/69, 39%) and the internet (24/69, 35%).

Wearable devices measured >1 biosignal in 88% (61/69) of the studies (Table 3). The most commonly measured biosignals were physical activity measures (eg, step counts, calories, distance, and metabolic rate; 62/69, 90%), followed by sleep measures (eg, duration and patterns; 53/69, 77%) and heart rate measures (eg, heart rate, heart rate variability, and interbeat interval; 32/69, 46%). The wearable devices in the included studies contained 18 different sensors, and those in approximately 64% (44/69) of the included studies contained >1 sensor. The most common sensor in the wearable devices were accelerometers (63/69, 91%), followed photoplethysmography sensors (31/69, 45%). Although the wearable devices in 67% (46/69) of the studies used an opportunistic approach to collect data (ie, an automatic approach without the user’s input), those in the rest of the studies (23/69, 33%) used both opportunistic and participatory approaches (ie, manual input by the user). The wearable devices in 55% (38/69) of the studies used a passive sensing method to collect data (ie, the sensor captures only signals that come from an object without the transmission of signals to it), whereas those in the remaining (31/69, 45%) studies used both a passive sensing approach and an active sensing approach (ie, the sensor emits signals or light to an object and then captures the reflected signals or light via a detector to measure the biosignal). Multimedia Appendix 6 [19-87] shows the features of the sensors of the wearable devices in each included study.

**Table 2:** Features of the wearable devices (WDs) the included studies focused on (N=69).

Features		Values, n (%)	References
**Target condition**			
	Depression	44 (64)	[19,20,23-28,30,32,34-38,42,43,46-53,57,59,60,62,64-71,73,77,79,82-84,86]
	Anxiety	17 (25)	[21,22,31,39-41,45,58,61,72,74,75,78,80,81,85,87]
	Anxiety and depression	8 (12)	[29,33,44,54-56,63,76]
**Status of WD^a^**			
	Commercial	63 (91)	[19-38,40-43,46-60,62-71,73-81,83-87]
	Noncommercial	7 (10)	[39,44,45,61,72,82,87]
**Name of WD^b^**			
	Actiwatch AW4 (Cambridge Neurotechnology)	17 (25)	[19,20,32,34-36,42,43,47,48,62,65,68-71,86]
	Fitbit series (Fitbit Inc)	13 (19)	[25,26,30,31,33,38,50,52,59,63,73,80,84]
	Empatica series (Empatica Inc)	7 (10)	[27,37,58,66,75,78,85]
	3-Space Sensor (Yost Labs)	4 (6)	[54-56,76]
	Muse	3 (4)	[21,22,58]
	Others	29 (42)	[23,24,28,29,39-41,44-46,49,51,53,57,58,60,61,64,67,72,74,77,79-83,85,87]
	Not reported	5 (7)	[39,44,45,61,72]
**Company of WD^b^**			
	Cambridge Neurotechnology	17 (25)	[19,20,32,34-36,42,43,47,48,62,65,68-71,86]
	Fitbit Inc	11 (16)	[25,26,30,31,33,38,50,52,59,63,73,80,84]
	Empatica Inc	7 (10)	[27,37,58,66,75,78,85]
	YEI Technology	4 (6)	[54-56,76]
	InteraXon	3 (4)	[21,22,58]
	Philips	3 (4)	[41,46,57]
	Others	27 (39)	[23,24,28,29,39,40,44,45,49,51,53,57,58,60,61,64,67,72,74,77,79-83,85,87]
	Not applicable	5 (7)	[39,44,45,61,72,82]
**Type of WD^b^**			
	Smart band	50 (72)	[21-26,28-31,33-40,42,44,45,47,50-59,61,63,66,69-76,78,79,81,84,85]
	Smartwatch	16 (23)	[19,20,32,41,43,46,48,49,60,62,65,67,68,77,83,86]
	Others (smart shirt, smart adhesive electrodes, smart headset, smart glasses, smart ring, and smart shirt)	5 (7)	[64,80,82,85,87]
**Placement^b^**			
	Wrist	57 (83)	[19,20,23-39,41-52,57-63,65-71,73-75,77-81,83-86]
	Head	7 (10)	[21,22,53,54,58,76,87]
	Waist	6 (9)	[28,54-56,72,76]
	Chest	4 (6)	[58,80,82,85]
	Others (ankle, arm, eyes, finger, hand, neck, and thigh)	1 (each) (1)	[27,39,40,64,87]
**Compatibility with OS^c,d^**			
	Windows (Corp)	52 (75)	[19-22,25-27,30-38,40-43,46-50,52,53,57-59,61-63,65-75,78,80,81,83-87]
	iOS (Apple Inc)	36 (52)	[21-31,33,37,38,50-53,58-60,63,64,66,73-75,77-81,83-85,87]
	Android	35 (51)	[21-23,25-28,30,31,33,37,38,40,50-53,58-60,63,64,66,73-75,77-81,83-85,87]
	Mac OS (Apple Inc)	27 (39)	[21,22,25-27,30,31,33,37,38,50,52,53,58,59,63,66,67,73-75,78,80,81,84,85,87]
	Linux	3 (4)	[21,22,58]
	Not reported	8 (12)	[39,44,45,54-56,76,82]
**Gateway^e^**			
	Smartphone	21 (30)	[23,25,26,29-31,33,38,40,50,52,59-61,63,64,73,79,80,83,84]
	PC	13 (19)	[25,26,30,31,33,38,50,52,59,63,73,80,84]
	Tablet	13 (19)	[25,26,30,31,33,38,50,52,59,63,73,80,84]
	Silmee L20 gateway	1 (1)	[79]
	Not reported	48 (70)	[19-22,24,27,28,32,34-37,39,41-49,51,53-58,62,65-72,74-78,81,82,85-87]
**Host^f^**			
	PC	46 (67)	[19-22,27,28,32,34-37,39-49,53-58,62,65-72,74-78,81,85-87]
	Server	30 (43)	[23,25-27,29-31,33,37,38,50,52,58-61,63,64,66,73-75,78-85]
	Smartphone	16 (23)	[21,22,24,27,37,51,53,58,66,74,75,77,78,81,85,87]
	Tablet	8 (12)	[21,22,53,58,74,77,81,87]
**Mode of data transfer^g^**			
	Bluetooth	41 (59)	[21-27,29-31,33,37,38,40,50-56,58-61,63,64,66,73-81,83-85,87]
	Docking station	27 (39)	[19,20,27,32,34-37,41-43,47-49,57,62,65-71,75,78,85,86]
	Internet	24 (35)	[23,25,26,29-31,33,38,40,50,52,54-56,59,61,63,64,73,76,79,80,83,84]
	Removable media	8 (12)	[39,44,45,54-56,76,82]
	Wired	8 (12)	[28,46,54-56,58,72,76]
	ANT+ (ANT Wireless)	1 (1)	[81]

^a^The number of studies does not add up, as 1 (1%) study has both commercial and noncommercial wearable devices.

^b^The number of studies does not add up, as several studies have used >1 wearable device.

^c^The number of studies does not add up, as several studies have used >1 wearable device, and many wearable devices are compatible with >1 operating system.

^d^OS: operating system.

^e^The number of studies does not add up, as several studies used >1 wearable device, and many wearable devices used >1 gateway.

^f^The number of studies does not add up, as several studies used >1 wearable device, and many wearable devices used >1 host.

^g^The number of studies does not add up, as several studies used >1 wearable device, and many wearable devices used >1 mode of data transfer.

**Table 3:** Features of the sensors of the wearable devices in the included studies (N=69).

Feature			Studies, n (%)		References
**Measured biosignals^a^**					
	Physical activity measures	62 (90)		[19,20,23-28,30-39,41-52,54-60,62-86]	
	Sleep measures	53 (77)		[19,20,23-27,30-38,41-43,46-52,57-60,62-71,73-75,77-86]	
	Heart rate measures	32 (46)		[23,26,27,29-31,33,37,38,40,50,51,58-61,63,64,66,72-75,77-83,85,87]	
	Skin temperature	12 (17)		[27,37,39,44,58,64,66,75,78,79,83,85]	
	Electrodermal activity	11 (16)		[27,37,40,58,61,66,72,75,78,83,85]	
	Light exposure	7 (10)		[28,41,46,49,57,77,83]	
	Electroencephalograph	5 (7)		[21,22,53,58,87]	
	Respiration measures	5 (7)		[40,64,72,80,82]	
	Audio	4 (6)		[39,44,54,83]	
	Electrocardiograph sensor	3 (4)		[40,80,85]	
	UV level	3 (4)		[64,79,83]	
	Skin humidity	2 (3)		[39,44]	
	Air pressure	2 (3)		[60,83]	
	Others (blood oxygen saturation and location)	1 (each) (1)		[40,81]	
**Sensors in the wearables^b^**					
	Accelerometer	63 (91)		[19,20,23-39,41-52,54-60,62-86]	
	PPG^c^ sensors	31 (45)		[23,26,27,29-31,33,37,38,40,50,51,58-61,63,64,66,72-75,77-81,83,85,87]	
	Thermometer	12 (17)		[27,37,39,44,58,64,66,75,78,79,83,85]	
	Gyroscope	12 (17)		[39,44,45,54-56,60,64,72,76,77,83]	
	Electroencephalograph sensor	11 (16)		[27,37,40,58,61,66,72,75,78,83,85]	
	Altimeter	10 (14)		[26,31,33,38,50,63,73,74,80,81]	
	Light sensors	7 (10)		[28,41,46,49,57,77,83]	
	Electrocardiograph sensor	5 (7)		[40,58,80,82,85]	
	Compass	5 (7)		[54-56,76,77]	
	Microphone	4 (6)		[39,44,54,83]	
	UV sensor	3 (4)		[64,79,83]	
	Barometer	2 (3)		[60,83]	
	Others (GPS, oximeter, and piezoelectric sensor)	1 (each) (1)		[40,81,83]	
**Sensing approach^d^**					
	Opportunistic	69 (100)		[19-87]	
	Participatory	23 (33)		[19,20,27,32,34-37,42,43,46-48,57,58,62,65,66,68-71,86]	
**Sensing type^e^**					
	Passive	38 (55)		[19-22,25,26,28,33,35-37,40,42-50,53-58,63,65,67-71,76,82,84,86]	
	Passive and Active	31 (45)		[23,24,26,27,29-32,34,38,39,41,51,52,59-62,64,66,72-75,77-81,83,85,87]	

^a^The number of studies does not add up, as several studies used >1 wearable device, and most wearable devices assess >1 biosignal.

^b^The number of studies does not add up, as several studies used >1 wearable device, and most wearable devices have >1 sensor.

^c^PPG: photoplethysmography.

^d^The number of studies does not add up, as several studies used >1 wearable device, and many wearable devices used >1 sensing approach.

^e^The number of studies does not add up, as several studies used >1 wearable device, and many wearable devices used >1 sensing type.

### Features of AI Algorithms

The included studies used AI for three clinical purposes: (1) diagnosing or screening for anxiety and depression (41/69, 59%), (2) monitoring symptoms or levels of anxiety and depression (15/69, 22%), and (3) predicting the occurrence or level of anxiety and depression in the future based on previous and current biosignals (13/69, 19%; Table 4). The included studies used only machine learning algorithms (46/69, 67%), only deep learning algorithms (7/69, 10%), or both machine learning and deep learning algorithms (16/69, 23%). The studies used these algorithms to solve classification problems (63/69, 91%), regression problems (11/69, 16%), and clustering problems (3/69, 4%). More than 50 different algorithms were used in the included studies; however, the most commonly used algorithm was random forest (36/69, 52%), followed by support vector machine (26/69, 38%), logistic regression (16/69, 23%), decision tree (16/69, 23%), extreme gradient boosting (11/69, 16%), and k-nearest neighbors (11/69, 16%). Multimedia Appendix 7 [19-87] shows the features of the AI algorithms used in each included study.

The included studies identified the ground truth based on 27 different tools, but the most common tool was Montgomery-Asberg Depression Rating Scale (MADRS; 17/69, 25%), followed by Patient Health Questionnaire-9 (12/69, 17%) and State-Trait Anxiety Inventory (8/69, 12%). The included studies used 7 different validation methods for the models. Approximately, 22% (15/69) of the included studies used >1 validation method (Table 4). The most commonly used validation method was k-fold cross-validation (33/69, 48%), followed hold-out cross-validation (25/69, 36%) and leave-one-out cross-validation (20/69, 29%). The included studies evaluated the performance of the models using 33 metrics. The most common metric used in the included studies was accuracy (50/69, 72%), followed by sensitivity (41/69, 59%), *F*_1_-score (30/69, 43%), specificity (28/69, 41%), precision (24/69, 35%), and area under the curve (22/69, 32%).

Approximately, 20% (14/69) of the included studies reported the data set size used for developing (ie, training and testing) the models (Table 5). The data set size ranged between 168 and 1,570,144 inputs, with an average of 168,023 (SD 428,843) inputs. The included studies used data sets from either closed sources (ie, collected by the authors of the study or obtained from previous studies; 50/69, 72%) or open sources (ie, public databases; 19/69, 28%). Depression was the most common data set obtained from open sources and used in the included studies (16/19, 84%). In 59% (41/69) of the studies, AI algorithms were developed using only data collected by wearable devices. Of the included studies, approximately 17% (12/69) developed AI algorithms using data collected by a combination of wearable devices and self-administered questionnaires (ie, self-reported data), approximately 13% (9/69) developed AI algorithms using data collected by a combination of wearable devices and nonwearable devices (eg, smartphones), and approximately 10% (7/69) developed AI algorithms using data collected by a combination of wearable devices, nonwearable devices, and self-administered questionnaires. The included studies used >50 categories of data to develop their models. Although 43% (30/69) of the studies used only 1 category of data to develop their models, the rest (39/69, 57%) of the studies used >1 category of data. The most common category of data used to develop the models was physical activity data (eg, step counts, calories, and metabolic rate; 53/69, 77%), followed by sleep data (eg, duration and patterns; 27/69, 39%), heart rate data (eg, heart rate, heart rate variability, and interbeat interval; 26/69, 38%), mental health measures (eg, depression level, anxiety level, stress level, and mood status; 14/69, 20%), location data (eg, latitude, longitude, percentage of time spent at home, and stationary time; 10/69, 14%), smartphone use data (eg, display on or off, charging activity, and the number of apps used; 10/69, 14%), and social interactions (eg, call and message logs; 10/69, 14%). The number of features used in the model development ranged from 2 to 5173. In approximately half (33/69, 48%) of the studies, the number of features was ≤10. Multimedia Appendix 8 [19-87] shows the features of the data used for AI development in each included study.

**Table 4:** Features of the artificial intelligence (AI) algorithms used in the included studies (N=69).

Feature			Studies, n (%)	References
**AI category**				
	ML^a^	46 (67)		[19,20,23,25,26,31,33,34,37,39,40,42,46,49-61,63-67,69-71,73,75-81,83,84,86,87]
	DL^b^	7 (10)		[24,29,32,44,47,62,82]
	ML and DL	16 (23)		[21,22,27,28,30,35,36,38,41,43,45,48,68,72,74,85]
**Problem-solving approaches^c^**				
	Classification	63 (91)		[19-36,38-58,60-65,67-73,75,76,78-82,84-87]
	Regression	11 (16)		[37,42,50,59,66,73,74,77,79,83,85]
	Clustering	3 (4)		[31,74,85]
**AI algorithm^d^**				
	Random forest	36 (52)		[19-23,26,27,30,33-38,41,43,45,46,49,51,53,59-61,64-66,68-71,77-79,81,86]
	Support vector machine	26 (38)		[19,20,23,27,30,31,35,38,40,41,49,53,55,56,58,60,61,64,67,72,75,77-80,87]
	Logistic regression	16 (23)		[19,21-23,25,28,30,38,46,49,51,55-57,61,64]
	Decision tree	16 (23)		[20,23,27,35,38,40,46,49,54-56,72,76,78,81]
	Extreme gradient boosting	11 (16)		[20,27,28,41,42,59,64,73,74,79,81]
	k-nearest neighbors	11 (16)		[23,27,35,38,40,41,55,56,64,78,87]
	AdaBoost	9 (13)		[25,30,35,37,59,68,77,81,84]
	Multilayer perceptron	8 (12)		[21,22,24,27,28,72,74,82]
	Convolutional neural network	7 (10)		[32,43-45,47,48,62]
	Gradient boosting	5 (7)		[25,27,45,59,77]
	Naive Bayes	5 (7)		[23,35,38,40,53]
	Others	28 (41)		[19,28-31,35-37,40,41,43-45,47,48,50-53,59,63,66,68,74,77,81,83,85]
**Aim of AI algorithm**				
	Diagnosis or screening	41 (59)		[19,21,22,28,32,35,36,38-40,43,46-49,51,53-57,61-63,65,67-71,73-76,78-80,82,83,85,87]
	Monitoring	15 (22)		[20,23,27,34,37,42,44,45,50,58,60,64,66,72,86]
	Prediction	13 (19)		[24-26,29-31,33,41,52,59,77,81,84]
**Ground truth assessment^e^**				
	MADRS^f^	17 (25)		[19,20,32,34-36,42,43,47,48,62,65,68-71,86]
	PHQ^g^-4, -8, and -9	13 (19)		[23,24,27,28,30,38,52,53,59,60,73,77,83]
	STAI^h^	8 (12)		[21,22,29,31,39,44,61,74]
	DSM^i^-IV and -5	6 (9)		[26,50,55,56,60,82]
	BDI-II^j^	4 (6)		[25,44,60,84]
	Others	26 (38)		[27,29,33,37,40,41,45,46,49-51,54,57,58,63,64,66,69,75,76,78,79,81,82,85,87]
	Not reported	3 (4)		[67,72,80]
**Validation approach^k^**				
	k-fold cross-validation	33 (48)		[21-24,27,30,32,34,35,37,38,40,41,45,47,51,52,60,62,63,66,68,69,73-75,78-83,87]
	Hold-out cross-validation	25 (36)		[26,28,29,31,32,34,37,44-46,48,49,51,60-62,66,67,70,71,74,81,82,84,86]
	Leave-one-out cross-validation	20 (29)		[20,25,32,33,36,37,42,43,45,50,53-56,58,59,75,76,84,85]
	Nested cross-validation	3 (4)		[19,64,77]
	External validation	1 (1)		[57]
	Time-series cross-validation	1 (1)		[64]
	Repeated random subsampling	1 (1)		[87]
	Not reported	3 (4)		[39,65,72]
**Performance measures^l^**				
	Accuracy	50 (72)		[20-29,31-33,35,36,38-40,42,43,46-49,51,53-56,60-64,67-71,73,75,76,78,79,81,82,84,86-88]
	Sensitivity	41 (59)		[19,21-23,26-28,32-36,38,41-43,46,47,51-54,56-58,60,62,64,65,67-73,79-81,84,86]
	*F*_1_-score	30 (43)		[19-22,25,27,28,32,33,35,36,38,44,46,47,50-52,60-64,67-69,72,80,81,84]
	Specificity	28 (41)		[19,21,26,32,34-36,41-43,46,47,51-54,56,58,62,65,67,70,71,73,79-81,86]
	Precision	24 (35)		[19,22,28,32,33,35,36,38,46,47,51,53,58,60,62,64,67,68,70-73,84,86]
	Area under the curve	22 (32)		[19,26,28,30,34,40,41,46,51,54-57,62,64,65,67,69,70,73,81,86]
	Mean absolute error	9 (13)		[21,22,48,59,66,73,77,79,83]
	Matthews correlation coefficient	9 (13)		[35,36,43,47,62,68,69]
	Cohen κ	7 (10)		[21,22,40,42,52,68,73]
	Root mean square error	6 (9)		[21,22,37,59,66,73]
	Balanced accuracy	6 (9)		[19,41,52,67,80,86]
	Receiver operating characteristic	6 (9)		[19,27,55,65,81,86]
	Correlation coefficient (*r*)	5 (7)		[42,66,74,79,83]
	Others	13 (19)		[22,40,50,52,53,57,59,71,73,74,77,85,86]

^a^ML: machine learning.

^b^DL: deep learning.

^c^The number of studies does not add up, as many studies have used >1 problem-solving approach.

^d^The number of studies does not add up, as many studies have used >1 AI algorithm.

^e^The number of studies does not add up, as many studies have used >1 tool to assess the ground truth.

^f^MADRS: Montgomery-Asberg Depression Rating Scale.

^g^PHQ: Patient Health Questionnaire.

^h^STAI: State-Trait Anxiety Inventory.

^i^DSM: Diagnostic and Statistical Manual of Mental Disorders.

^j^BDI-II: Beck Depression Inventory-Second Edition.

^k^The number of studies does not add up, as many studies have used >1 validation approach.

^l^The number of studies does not add up, as most studies used >1 performance measure.

**Table 5:** Features of the data used for artificial intelligence (AI) development in the included studies (N=69).

Feature		Values	References
Data set size, mean (SD; range)		168,022.5 (428,843.2; 168-1,570,144)	[22,23,28,37,41,44,45,51,58,60-62,70,73]
**Data set source, n (%)**			
	Open	19 (28)	[19,20,28,31,32,34,36,42,43,47,48,62,65,68-71,74,86]
	Closed	50 (72)	[21-27,29,30,33,35,37-41,44-46,49-61,63,64,66,67,72,73,75-85,87]
**Data types, n (%)**			
	WD^a^ based	41 (59)	[20-22,27,29,31-36,38,39,41-48,53-56,58,61,62,65,67,69-71,73,75,76,78-80,82,87]
	WD based and self-reported	12 (17)	[19,26,28,30,49,51,52,57,68,81,85,86]
	WD based and non-WD based	9 (13)	[23,25,40,50,59,66,72,74,84]
	WD based, non-WD based, and self-reported	7 (10)	[24,37,60,63,64,77,83]
**Data input to AI algorithm^b^, n (%)**			
	Physical activity data	53 (77)	[19,20,23-27,30-32,34-38,41-51,54-57,59,60,62-74,76,77,79,81,83-86]
	Sleep data	27 (39)	[23-26,30,33,37,38,41,46,49-52,57,59,60,63,64,66,73,74,77,79,81,83,84]
	Heart rate data	26 (38)	[23,26,27,29-31,40,50,51,58-61,63,64,66,72,75,77-81,83,85,87]
	Mental health measures	14 (20)	[24,26,30,37,46,49,50,52,57,60,64,77,81,85]
	Social interaction data	10 (14)	[23-25,37,59,60,66,72,83,84]
	Location data	10 (14)	[23,25,37,50,59,64,66,74,83,84]
	Smartphone use data	10 (14)	[23,25,37,59,60,64,66,74,83,84]
	Electrodermal activity data	10 (14)	[27,37,40,58,61,66,72,75,78,85]
	Skin temperature data	5 (7)	[27,75,78,79,85]
	Demographic data	5 (7)	[30,52,57,68,85]
	Electroencephalograph data	4 (6)	[21,22,53,87]
	Light exposure	4 (6)	[26,46,60,79]
	Audio data	4 (6)	[39,44,54,85]
	Others	17 (25)	[24,28,30,37,49,52,57,60,63,66,72-74,77,81,82,85]
**Number of features^c^, n (%)**			
	1-10	33 (48)	[19,21-25,27,34-40,43,46,47,50,54-58,67,69-72,75,78,82,83,87]
	11-20	16 (23)	[23,26,28,30,33,45,48,51-53,57,61,68,72,76,86]
	21-30	6 (9)	[44,52,60,63,73,85]
	31-40	6 (9)	[23,34,38,50,66,73]
	41-50	6 (9)	[23,41,64,73,77,80]
	>50	8 (12)	[23,27,59,73,74,79,81,84]
	Not reported	8 (12)	[20,29,31,32,42,49,62,65]

^a^WD: wearable device.

^b^The number of studies does not add up, as many studies used >1 data input.

^c^The number of studies does not add up, as several studies used various numbers of features.

## Discussion

### Principal Findings

This scoping review aimed at exploring the features of AI and wearable devices used for anxiety and depression. In this review, approximately two-thirds of the studies used wearable AI for depression, whereas the remaining studies used it for anxiety. This may be attributed to the ability of wearables to collect biosignals related to the symptoms of depression and anxiety. More specifically, it is well known that depression is associated with a decrease in activity and changes in sleep behaviors [13,89,90], which can be objectively measured using wearable devices. Furthermore, the analysis of depression symptoms does not rely on highly accurate data; that is, general trends are sufficient to provide indications. By contrast, anxiety is usually associated with heart rate variability [91]. Although wearable devices can have an acceptable heart rate accuracy [92], the quality of one device is different from that of another [93]. Moreover, monitoring the heart rate without contextual information might be misleading because multiple factors impact the heart rate; thus, detecting anxiety based on only objective biosignals is questionable. Combining the data from wearable devices with additional data sources is crucial. So far, only a few studies included in this review were based on a combination of data from different sources (ie, wearable devices, nonwearable devices, and self-administered questionnaires).

In this review, the most frequent application of wearable AI was in the diagnosis of or screening for anxiety and depression. A similar result was reported in 2 previous reviews, which showed that most studies focused on using wearables for diagnostic purposes [10,13]. Although wearable AI can be used for interventional and treatment purposes (eg, personalized mindfulness, meditation, and biofeedback therapy [14]), none of the systems in the included studies were used for such purposes. This may be attributed to the lack of evidence on the effectiveness of wearable AI in improving anxiety and depression.

Smart bands worn on the wrist were the most common type of wearable device used in the studies. This has been indicated in previous reviews as well [10,13,14]. This can be attributed to the fact that wrist-worn wearable devices are less distractive and less obtrusive, easy to use, and more stylish and familiar to most people. According to Hunkin et al [94], such features are crucial for users’ acceptance and use of wearable devices.

The most commonly used category of data for model development was physical activity data, followed by sleep data and heart rate data. This is expected given that depression and anxiety are associated with physical activity [13,89,90], sleep patterns [13,95,96], and heart rate [91]. In addition, as this review demonstrated, these are the most common biosignals measured by commercial wearable devices.

Surprisingly, more than half of the studies considered only data from wearables in their AI algorithms. However, wearables cannot detect all the symptoms relevant to anxiety and depression for 2 reasons. First, wearable devices cannot detect several physiological data, such as weight loss or gain and changes in appetite [13]. Second, wearable devices cannot evaluate subjective symptoms such as social interaction, medical history, and lifestyle changes [13]. It might be questioned whether research has started to overrely on the diagnostic and predictive power of data from only wearable devices.

Approximately, one-fourth of the studies relied on a data set called Depresjon [35] to develop their models. Depresjon is a freely available data set that contains data related to motor activity measured using an actigraph watch worn on the wrist (Actiwatch AW4) [35]. The data set also contains data related to depression levels assessed using MADRS [35]. This explains why the most common wearable device used in the included studies was Actiwatch AW4 and why MADRS was the most frequently used tool to assess the ground truth.

Regarding the target population, we must recognize that most studies addressed individuals aged between 18 and 65 years. Global statistics show that the that the incidence of depression and anxiety is slightly higher in age groups between 15-64 years than adult aged ≥65 years [1]. This might explain why the studies mainly targeted the age group of 18 to 65 years. Another explanation might be that wearables are more popular among adults in that age group.

This review showed that k-fold cross-validation was the most frequently used validation method. This can be attributed to several factors. First, in comparison with hold-out cross-validation, k-fold cross-validation is prone to less variation, as each observation is used for both training and testing. Second, the training set in k-fold cross-validation is larger than that in hold-out cross-validation; therefore, k-fold cross-validation has reduced bias and reduced overestimation of test error. Finally, k-fold cross-validation is computationally less expensive than leave-one-out cross-validation, as the algorithm needs to rerun only k times (usually ≤10).

### Research and Practical Implications

The performance of wearable AI in diagnosing, monitoring, and predicting anxiety and depression was not assessed in this review. Systematic reviews and meta-analyses are needed to examine the performance of wearable AI devices. Future studies should also compare the performance of different wearable devices (eg, Fitbit vs Empatica), worn at different locations (eg, wrist, chest, and waist), and using different data types (eg, wearable-based data vs wearable-based data and self-reported data). Conducting systematic reviews of such studies can help researchers, developers, and wearable device companies identify the most significant features and powerful AI algorithms for diagnosing, monitoring, and predicting anxiety and depression.

AI research highly depends on the available data sets. However, when only 1 data set is exploited by researchers, no conclusions regarding the generalizability of study results can be drawn. Therefore, we recommend that researchers (1) publish their data sets in open databases after ensuring participants’ privacy and confidentiality and (2) exploit different data sets available in open databases.

This review found a lack of AI-based wearable devices used for treatment purposes, although wearable AI can be used to provide many interventions for anxiety and depression, such as personalized mindfulness, meditation, and biofeedback therapy. Technology companies should invest more in wearable AI devices for the treatment of anxiety and depression. Researchers should also assess the effectiveness of such technologies in improving anxiety and depression.

The ground truth of mental states (anxiety or depression) in the included studies was identified using 27 different tools. Although most of these tools have been validated extensively, they do not usually include physiological biomarkers (eg, physical activity, heart rate, electrodermal activity, respiratory rate, and electroencephalogram). This brings into question the validity and reliability of drawing conclusions about mental states (anxiety or depression) based on physiological biomarkers when the ground truth of mental states is assessed using subjective questionnaires. Accordingly, the performance of AI-based wearable devices will be underestimated.

Although the current studies have shown that wearable AI can be used for monitoring symptoms or levels of anxiety and depression, continuous tracking of physiological biomarkers could trigger emotional instability and ruminative thinking [97]. Although wearable AI can approximate mental states (eg, feeling nervous, anxious, or on edge) through heart rate and other variables, it could provide many false positives, thereby exacerbating or increasing the anxiety or depression of an individual. The abovementioned downsides of wearable AI should be considered and mitigated before developing AI-based wearables in the future. More research is needed on the use of wearable devices and individuals’ emotional and behavioral responses to the automated feedback from wearable devices.

Wearable AI can help individuals conduct prescreening assessments of mental health and well-being without an initial hospital or clinical encounter. The individual could be notified through the wearable device, smartphone, or desktop application about their mental health status, which would encourage them to visit a mental health and well-being professional. Such prescreening feedback from wearables may help reduce mental health stigma and allow a higher number of individuals to seek help from a mental health professional.

The quality of the data, whether obtained from open sources or generated by wearable devices, should be emphasized. To do so, there is a need for more practical standards for wearable device development that ensure accurate measurement of different signals generated by wearable devices to improve algorithmic performance.

### Limitations

This review excluded many studies that focused on nonwearable devices, handheld devices (eg, mobile phones), near-body wearable devices, in-body wearable devices (eg, implants), wearable devices connected to nonwearable devices using wires, and wearable devices that can be placed on users only by experts. Therefore, our findings may not be generalizable to contexts in which such excluded devices are applied. Owing to practical constraints, we included only studies published in the English language. We also restricted our search to studies published from 2015 onward, given that this is a fast-growing field and, thereby, studies published before 2015 can be deemed outdated. Consequently, it is likely that we missed some studies published in other languages or before 2015. Another limitation of this review is that we cannot comment on the performance of wearable AI in diagnosing, monitoring, and predicting anxiety and depression and the importance of features and variables, as this is beyond the scope of this review and requires systematic reviews, wherein the quality of the evidence and risk of bias are assessed.

### Conclusions

Wearable AI can offer great promise in providing mental health services related to anxiety and depression. Wearable AI can be used by individuals for the prescreening assessment of anxiety and depression. Further reviews are needed to statistically synthesize the results of studies on the performance and effectiveness of wearable AI. More studies are needed on the use of wearable devices and individuals’ emotional and behavioral responses to the automated feedback from wearable devices. Given its potential, technology companies should invest more in wearable AI for the treatment of anxiety and depression. The downsides of wearable AI devices (eg, false positive alerts and triggering emotional instability and ruminative thinking) should be considered and mitigated before developing them in the future.

## Appendix

Multimedia Appendix 1 PRISMA-ScR (Preferred Reporting Items for Systematic Reviews and Meta-Analyses Extension for Scoping Reviews) checklist.

Multimedia Appendix 2 Search strategy.

Multimedia Appendix 3 Data extraction form.

Multimedia Appendix 4 Characteristics of each included study.

Multimedia Appendix 5 Features of wearable devices.

Multimedia Appendix 6 Features of the sensors of wearable devices.

Multimedia Appendix 7 Features of artificial intelligence algorithms.

Multimedia Appendix 8 Features of the data used in artificial intelligence algorithms.

## Abbreviations

AI: artificial intelligence
MADRS: Montgomery-Asberg Depression Rating Scale
PRISMA-ScR: Preferred Reporting Items for Systematic Reviews and Meta-Analyses Extension for Scoping Reviews

## Data Availability

The data supporting the findings of this study are available from the corresponding author upon reasonable request.
