# Correction: Wearable Artificial Intelligence for Anxiety and Depression: Scoping Review

**DOI:** 10.2196/46233

**Published:** 2023-02-07

**Authors:** Alaa Abd-alrazaq, Rawan AlSaad, Sarah Aziz, Arfan Ahmed, Kerstin Denecke, Mowafa Househ, Faisal Farooq, Javaid Sheikh

**Affiliations:** 1 AI Center for Precision Health Weill Cornell Medicine-Qatar Doha Qatar; 2 Institute for Medical Informatics, Bern University of Applied Science Bern Switzerland; 3 Division of Information and Computing Technology, College of Science and Engineering, Hamad Bin Khalifa University, Qatar Foundation Doha Qatar; 4 Qatar Computing Research Institute, Hamad bin Khalifa University Doha Qatar

In “Wearable Artificial Intelligence for Anxiety and Depression: Scoping Review” (J Med Internet Res 2023;25:e42672) the authors noted one error.

In Multimedia Appendix 8, the columns reporting the information extracted did not match the studies in the first column. An amended version will replace the erroneous version to realign this information.

The correction will appear in the online version of the paper on the JMIR Publications website on February 7, 2023 together with the publication of this correction notice. Because this was made after submission to PubMed, PubMed Central, and other full-text repositories, the corrected article has also been resubmitted to those repositories.

## Appendix

Multimedia Appendix 8 Features of the data used in artificial intelligence algorithms.

