# Supplementary material for: Wearable Artificial Intelligence for Anxiety and Depression: Scoping Review
Source: J Med Internet Res. 2023 Jan 19;25:e42672. doi: 10.2196/42672 (PMC9896355; doi:10.2196/42672)
Supplement: Multimedia Appendix 3 [file jmir_v25i1e42672_app3.docx]

| **Extracted data** | **Definition** |
| --- | --- |
| **Study Characteristics** |  |
| Author | The first author of the study. |
| Year of publication | The year in which the study was published. |
| Country of publication | The country where the study was published. |
| Type of publication | The venue where the study was published: peer-reviewed journal articles, book chapters, dissertations, or conference proceedings |
| **Wearable devices characteristics** |  |
| Targeted health condition | What is the health condition/disease that the AI-based wearable device targeted (depression and\or anxiety)? |
| Name of the wearable device | What is the name of the wearable device (e.g., Fitbit, Empatica, ApplyWatch, ActiWatch, etc..)? |
| Company | What is the company name of the wearable device? |
| Status of the wearable device | Is the wearable device a prototype (non-commercial) or is it an already available commercial device (e.g., Fitbit, apple watch)? |
| Type of the wearable device | What is the type of wearable device (e.g., smart band, smart watch, smart glasses, smart clothes, smart socks, smart shoes, etc)? |
| Placement of the wearable device | Where the wearable device is worn during the experiment in paper or normally (wrist, chest, head, ears, forehead, eyes, fingers, foot, etc..)? |
| Operating systems | What are the operating systems that the wearable device is compatible with (Android, IOS, Windows, etc..)? |
| Gateway device | What device is used as a gateway to transfer the collected data from the wearable to the main host (smartphone, tablet, database server, PC)? |
| Host device | What is the end gate device that the wearable is synchronized with/ that stores data collected by the wearable device (smartphone, tablet, database server, PC)? |
| Mode of data transfer | How data is transferred from the wearable device to the host device (Bluetooth, Internet (WiFi or mobile network), Wired, Removable media (SD card, CD, etc..), Airdrop)? |
| Measured biosignals | What are the biosignals measured by the wearable device (heart rate, EEG, ECG, step counts, body temperature, blood pressure, etc..)? |
| Sensors | What are the sensors embedded in the wearable device (Photoplethysmogram, accelerator, Gyroscope)? |
| Sensing approach | Does the wearable device collect the data with the user’s input (i.e., manually adding data; participatory approach) or without the user’s input (i.e., automatic mechanisms for collecting data; opportunistic approach)? |
| Sensing type | What is the sensing method that the sensors used to collect the data: active (i.e., the sensor transmits signals to an object, then capture them) or passive (i.e., the sensor captures only signals that come from an object without transmission of signals to it)? |
| **AI** **Characteristics** |  |
| AI categories | What is the category of each algorithm used in the study (Machine learning, deep learning, transfer learning, reinforcement learning, etc.)? |
| Problem solving approaches | What is the problem-solving approach that the algorithm follows (Classification, regression)? |
| AI algorithm used | What are the main AI algorithms/models (e.g., RF, SVM, ANN, CNN, RNN, DNN, k-NN, MLP, DBN, DBM, DPN BN, CRT, DT, LASSO, LR, MFA, MLR, MDL, NB, NN, NSC, RBFN) used in the paper? |
| Aim of AI algorithm | What was the algorithm used for (diagnosis, screening, monitoring, treatment, prevention, etc.))? |
| Dataset size | What is the dataset size used for developing (training & testing) the algorithm? |
| Number of participants | What is the number of participants from which the data was collected? |
| Mean age (range) | What is the mean/range age of the participants? |
| Male percentage | What is the male percentage of the participants? |
| Health condition | What is the health condition of the participants? |
| Data sources | What is the source of data that was used for developing the algorithms (open source or closed source)? |
| Type of data | What is the type of data (e.g., WD-based data, self-reported data, non-WD-based data) that was used for developing the algorithm? |
| Data input | What is the data that was used for developing the algorithm? |
| Ground truth | How the actual status (e.g., diagnosis) of the user was confirmed (questionnaire (PHQ-9), interview, test, etc..)? |
| Type of validation | What is the approach that was used to validate the developed algorithm (e.g., Training-test split, K-fold cross-validation, Nested Cross-Validation, Leave One Out cross-validation, Apparent validation, external validation)? |
| Performance measures used | What are the measures used to assess the performance of the algorithm (accuracy, sensitivity (recall), specificity, precision, AUC, etc...)? |
