# Supplementary material for: Wearable Artificial Intelligence for Anxiety and Depression: Scoping Review
Source: J Med Internet Res. 2023 Jan 19;25:e42672. doi: 10.2196/42672 (PMC9896355; doi:10.2196/42672)
Supplement: Multimedia Appendix 4 [file jmir_v25i1e42672_app4.docx]

**Multimedia Appendix 4: Characteristics of each included study**

| Study [Ref] | Year | Publication type | Country | Sample size | Mean age | Age range | Female % | Participants’ health condition |
| --- | --- | --- | --- | --- | --- | --- | --- | --- |
| Adamczyk [19] | 2021 | Journal article | Poland | 55 | 40.1 | NR | 55 | Depression, healthy |
| Aminifar [20] | 2021 | Conference Paper | Norway | 55 | 40.1 | NR | 55 | Depression, healthy |
| Arsalan [21] | 2020 | Conference Paper | Pakistan | 28 | NR | 18-40 | 53.6 | General |
| Arsalan [22] | 2021 | Journal article | Pakistan | 65 | 27.5 | NR | 49 | General |
| Bai [23] | 2021 | Journal article | China | 261 | NR | 18-60 | NR | Depression |
| Bennett [24] | 2017 | Conference Paper | United States | 8 | NR | NR | NR | Depression |
| Chikersal [25] | 2021 | Journal article | United States | 138 | NR | NR | NR | General |
| Cho [26] | 2019 | Journal article | Korea | 55 | 25.9 | 21-31 | 49.1 | Depression, bipolar |
| Choi [27] | 2021 | Journal article | Korea | 1552 | 42.1 | NR | 55 | General |
| Choi [28] | 2022 | Journal article | Korea | 14 | 76 | 65-86 | 85.7 | General |
| Coutts [29] | 2020 | Journal article | United Kingdom | 668 | 21.9 | 18-69 | 71.2 | General |
| Dai [30] | 2022 | Journal article | United States | 89 | 47.1 | NR | 76.4 | Depression |
| Feng [31] | 2020 | Conference Paper | United States | 97 | NR | NR | 74 | General |
| Frogner [32] | 2019 | Conference Paper | Norway | 55 | 40.1 | NR | 55 | Depression, healthy |
| Fukuda [33] | 2020 | Conference Paper | Japan | 60 | NR | NR | NR | General |
| Galvan-Tejada [34] | 2019 | Journal article | Mexico | 55 | 40.1 | NR | 55 | Depression, healthy |
| Garcia-Ceja [35] | 2018 | Conference Paper | Norway | 55 | 40.1 | NR | 55 | Depression, healthy |
| Garcia-Ceja [36] | 2018 | Conference Paper | Norway | 55 | 40.1 | NR | 55 | Depression, healthy |
| Ghandeharioun [37] | 2017 | Conference Paper | United States | 12 | 37 | 20-73 | 75 | Depression |
| Griffiths [38] | 2022 | Journal article | United Kingdom | 17 | 46.8 | 21-69 | 79 | Depression |
| Gu [39] | 2017 | Journal article | China | 22 | NR | NR | NR | General |
| Ihmig [40] | 2020 | Journal article | Germany | 57 | NR | 18-40 | NR | Arachnophobia |
| Jacobson [41] | 2019 | Journal article | United States | 55 | 40.1 | NR | 55 | Depression, healthy |
| Jacobson [42] | 2021 | Journal article | United States | 265 | 44.3 | 25-72 | 58.1 | General |
| Jakobsen [43] | 2020 | Journal article | Norway | 55 | 40.1 | NR | 55 | Depression, healthy |
| Jin [44] | 2020 | Journal article | China | 60 | NR | 18-26 | 50 | General |
| Khan [45] | 2021 | Journal article | Pakistan | 10 | NR | 20-50 | 90 | General |
| Kim [46] | 2019 | Journal article | Korea | 47 | 78 | NR | 94 | Depression |
| Kulam [47] | 2019 | Thesis | Norway | 55 | 40.1 | NR | 55 | Depression, healthy |
| Kumar [48] | 2022 | Journal article | United Kingdom | 55 | 40.1 | NR | 55 | Depression, healthy |
| Llamocca [49] | 2021 | Journal article | Spain | 17 | NR | NR | NR | Bipolar |
| Lu [50] | 2018 | Journal article | United States | 103 | NR | 18-25 | 76.7 | Depression, healthy |
| Mahendran [51] | 2019 | Journal article | India | 450 | 40 | NR | NR | Mood swings |
| Makhmutova [52] | 2022 | Journal article | Switzerland | 4036 | 36.8 | 18-85 | 73.7 | General |
| Mallikarjun [53] | 2020 | Journal article | India | 86 | NR | NR | 100 | General |
| McGinnis [54] | 2021 | Journal article | United States | 164 | 5.7 | 4-8 | NR | Internalizing disorders, healthy |
| McGinnis [55] | 2018 | Conference Paper | United States | 59 | 5.3 | 3-7 | 57 | Internalizing disorders, healthy |
| McGinnis [56] | 2019 | Journal article | United States | 59 | 5.3 | 3-7 | 57 | Internalizing disorders, healthy |
| Minaeva [57] | 2020 | Journal article | Netherlands | 179 | 46.5 | NR | 64 | Depression, healthy |
| Miranda [58] | 2017 | Journal article | Mexico | 10 | 24.7 | 23-26 | 50 | General |
| Mullick [59] | 2022 | Journal article | United States | 55 | 15.5 | NR | 77.2 | Depression |
| Narziev [60] | 2020 | Journal article | Korea | 20 | NR | NR | NR | Depression, healthy |
| Nath [61] | 2021 | Journal article | United States | 41 | 73.4 | 60-80 | 63.4 | General |
| Nguyen [62] | 2021 | Conference Paper | Taiwan | 55 | 40.1 | NR | 55 | Depression, healthy |
| Nishimura [63] | 2022 | Conference Paper | Japan | 100 | 42.1 | NR | 37 | General |
| Opoku Asare [64] | 2022 | Journal article | Finland | 54 | 43 | 24-68 | 55.6 | General |
| Pacheco-Gonzalez [65] | 2019 | Journal article | Mexico | 55 | 40.1 | NR | 55 | Depression, healthy |
| Pedrelli [66] | 2020 | Journal article | United States | 31 | 33.7 | 19-73 | 74 | Depression |
| Qian [67] | 2019 | Conference Paper | Japan | 83 | 38.4 | 22-58 | 2.4 | Depression, healthy |
| Raihan [68] | 2021 | Conference Paper | Bangladesh | 55 | 40.1 | NR | 55 | Depression, healthy |
| Rodríguez-Ruiz [69] | 2020 | Journal article | Mexico | 55 | 40.1 | NR | 55 | Depression, healthy |
| Rodríguez-Ruiz [70] | 2020 | Journal article | Mexico | 55 | 40.1 | NR | 55 | Depression, healthy |
| Rodríguez-Ruiz [71] | 2022 | Journal article | Mexico | 109 | 40.8 | NR | 48.8 | Depression, healthy, schizophrenia |
| Rother [72] | 2019 | Conference Paper | United Kingdom | 823 | NR | NR | NR | General |
| Rykov [73] | 2021 | Journal article | Singapore | 267 | 33 | 21-64 | 63.7 | General |
| Saha [74] | 2021 | Journal article | United States | 754 | 34.9 | 20-68 | NR | General |
| Šalkevicius [75] | 2019 | Journal article | Lithuania | 30 | 27.5 | 21-34 | 43.3 | Glossophobia, healthy |
| Scism [76] | 2020 | Thesis | United States | 63 | 5.2 | NR | 57 | Internalizing disorders, healthy |
| Shah [77] | 2021 | Journal article | United States | 14 | 21.6 | NR | 71.4 | Depression |
| Shaukat-Jali [78] | 2021 | Journal article | United Kingdom | 12 | 19.8 | NR | 58 | Social anxiety |
| Tazawa [79] | 2020 | Journal article | Japan | 86 | 60.2 | NR | 46.5 | Depression, healthy |
| Tiwari [80] | 2019 | Conference Paper | United States | 196 | 38.6 | NR | 66.3 | General |
| Tsai [81] | 2022 | Journal article | Taiwan | 59 | NR | 20-74 | 61 | Panic disorders |
| Valenza [82] | 2015 | Journal article | Italy | 8 | NR | NR | NR | Bipolar |
| Wang [83] | 2018 | Conference Paper | United States | 83 | 20.1 | NR | 51.8 | General |
| Xu [84] | 2019 | Journal article | United States | 350 | NR | NR | NR | General |
| Yadav [85] | 2019 | Conference Paper | United States | 55 | 21 | NR | 41.8 | General |
| Zanella-Calzada [86] | 2019 | Journal article | Mexico | 55 | 40.1 | NR | 55 | Depression, healthy |
| Zheng [87] | 2016 | Journal article | Hong Kong | 20 | NR | 18-23 | NR | General |
| Not reported, PC: Personal computer | | | | | | | | |
