# Supplementary material for: Wearable Artificial Intelligence for Anxiety and Depression: Scoping Review
Source: J Med Internet Res. 2023 Jan 19;25:e42672. doi: 10.2196/42672 (PMC9896355; doi:10.2196/42672)
Supplement: Multimedia Appendix 5 [file jmir_v25i1e42672_app5.docx]

**Multimedia Appendix 5: Features of wearable devices**

| Study [Ref] | Targeted health condition | Status of WD | Name of WD | Company | Type of WD | Placement of WD | Compatibility of Operating systems | Gateway | Host device | Mode of Data transfer |
| --- | --- | --- | --- | --- | --- | --- | --- | --- | --- | --- |
| Adamczyk [19] | Depression | Commercial | Actiwatch AW4 | Cambridge Neurotechnology | Smartwatch | Wrist | Windows | NR | PC | Docking station |
| Aminifar [20] | Depression | Commercial | Actiwatch AW4 | Cambridge Neurotechnology | Smartwatch | Wrist | Windows | NR | PC | Docking station |
| Arsalan [21] | Anxiety | Commercial | MUSE | InteraXon | Smart band | Head | Android, iOS, Linux, Mac OS, Windows | NR | PC, smartphone, tablet | Bluetooth |
| Arsalan [22] | Anxiety | Commercial | MUSE | InteraXon | Smart band | Head | Android, iOS, Linux, Mac OS, Windows | NR | PC, smartphone, tablet | Bluetooth |
| Bai [23] | Depression | Commercial | Mi Band 2 | Xiaomi Corporation | Smart band | Wrist | Android, iOS | Smartphone | Server | Bluetooth, Internet |
| Bennett [24] | Depression | Commercial | Jawbone UP | Jawbone | Smart band | Wrist | iOS | NR | Smartphone | Bluetooth |
| Chikersal [25] | Depression | Commercial | Fitbit Flex 2 | Fitbit Inc | Smart band | Wrist | Android, iOS, Mac OS, Windows | PC, smartphone, tablet | Server | Bluetooth, Internet |
| Cho [26] | Depression | Commercial | Fitbit Charge HR, Fitbit Charge 2 | Fitbit Inc | Smart band | Wrist | Android, iOS, Mac OS, Windows | PC, smartphone, tablet | Server | Bluetooth, Internet |
| Choi [27] | Depression | Commercial | ActiGraph GT3X | Actigraph Corp | Smart band | Ankle, thigh, waist, wrist | Android, iOS | NR | PC | Wired |
| Choi [28] | Depression | Commercial | Empatica E4 | Empatica | Smart band | Wrist | Android, iOS, Mac OS, Windows | NR | PC, server, smartphone | Bluetooth, docking station |
| Coutts [29] | Anxiety, depression | Commercial | Biobeam | BioBeats Group Ltd | Smart band | Wrist | iOS | Smartphone | Server | Bluetooth, Internet |
| Dai [30] | Depression | Commercial | Fitbit Alta HR | Fitbit Inc | Smart band | Wrist | Android, iOS, Mac OS, Windows | PC, smartphone, tablet | Server | Bluetooth, Internet |
| Feng [31] | Anxiety | Commercial | Fitbit Charge 2 | Fitbit Inc | Smart band | Wrist | Android, iOS, Mac OS, Windows | PC, smartphone, tablet | Server | Bluetooth, Internet |
| Frogner [32] | Depression | Commercial | Actiwatch AW4 | Cambridge Neurotechnology | Smartwatch | Wrist | Windows | NR | PC | Docking station |
| Fukuda [33] | Anxiety, depression | Commercial | Fitbit Charge 3 | Fitbit Inc | Smart band | Wrist | Android, iOS, Mac OS, Windows | PC, smartphone, tablet | Server | Bluetooth, Internet |
| Galvan-Tejada [34] | Depression | Commercial | Actiwatch AW4 | Cambridge Neurotechnology | Smart band | Wrist | Windows | NR | PC | Docking station |
| Garcia-Ceja [35] | Depression | Commercial | Actiwatch AW4 | Cambridge Neurotechnology | Smart band | Wrist | Windows | NR | PC | Docking station |
| Garcia-Ceja [36] | Depression | Commercial | Actiwatch AW4 | Cambridge Neurotechnology | Smart band | Wrist | Windows | NR | PC | Docking station |
| Ghandeharioun [37] | Depression | Commercial | Empatica E4 | Empatica | Smart band | Wrist | Android, iOS, Mac OS, Windows | NR | PC, server, smartphone | Bluetooth, docking station |
| Griffiths [38] | Depression | Commercial | Fitbit | Fitbit Inc | Smart band | Wrist | Android, iOS, Mac OS, Windows | PC, smartphone, tablet | Server | Bluetooth, Internet |
| Gu [39] | Anxiety | Non-commercial | NR | NA | Smart band | Neck, wrist | NR | NR | PC | Removable media |
| Ihmig [40] | Anxiety | Commercial | BITalino, Rythem+ | PLUX Biosignals, Scosche | Smart band | Arm, hand | Android, Windows | Smartphone | PC | Bluetooth, Internet |
| Jacobson [41] | Depression | Commercial | Actiwatch AW4 | Cambridge Neurotechnology | Smart band | Wrist | Windows | NR | PC | Docking station |
| Jacobson [42] | Anxiety | Commercial | Mini Mitter Actiwatch | Philips | Smartwatch | Wrist | Windows | NR | PC | Docking station |
| Jakobsen [43] | Depression | Commercial | Actiwatch AW4 | Cambridge Neurotechnology | Smartwatch | Wrist | Windows | NR | PC | Docking station |
| Jin [44] | Anxiety, depression | Non-commercial | NR | NA | Smart band | Wrist | NR | NR | PC | Removable media |
| Khan [45] | Anxiety | Non-commercial | NR | NA | Smart band | Wrist | NR | NR | PC | Removable media |
| Kim [46] | Depression | Commercial | Actiwatch Spectrum PRO | Philips | Smartwatch | Wrist | Windows | NR | PC | Wired |
| Kulam [47] | Depression | Commercial | Actiwatch AW4 | Cambridge Neurotechnology | Smart band | Wrist | Windows | NR | PC | Docking station |
| Kumar [48] | Depression | Commercial | Actiwatch AW4 | Cambridge Neurotechnology | Smartwatch | Wrist | Windows | NR | PC | Docking station |
| Llamocca [49] | Depression | Commercial | GENEActiv | Activinsights | Smartwatch | Wrist | Windows | NR | PC | Docking station |
| Lu [50] | Depression | Commercial | Fitbit Charge HR | Fitbit Inc | Smart band | Wrist | Android, iOS, Mac OS, Windows | PC, smartphone, tablet | Server | Bluetooth, Internet |
| Mahendran [51] | Depression | Commercial | Mi Band 3 | Xiaomi Corporation | Smart band | Wrist | Android, iOS | NR | Smartphone | Bluetooth |
| Makhmutova [52] | Depression | Commercial | Fitbit | Fitbit Inc | Smart band | Wrist | Android, iOS, Mac OS, Windows | PC, smartphone, tablet | Server | Bluetooth, Internet |
| Mallikarjun [53] | Depression | Commercial | MindWave Mobile | Neurosky | Smart band | Head | Android, iOS, Mac OS, Windows | NR | PC, smartphone, tablet | Bluetooth |
| McGinnis [54] | Anxiety, depression | Commercial | 3-Space Sensor | YEI Technology | Smart band | Head, waist | NR | NR | PC | Bluetooth, Internet, removable media, wired |
| McGinnis [55] | Anxiety, depression | Commercial | 3-Space Sensor | YEI Technology | Smart band | Waist | NR | NR | PC | Bluetooth, Internet, removable media, wired |
| McGinnis [56] | Anxiety, depression | Commercial | 3-Space Sensor | YEI Technology | Smart band | Waist | NR | NR | PC | Bluetooth, Internet, removable media, wired |
| Minaeva [57] | Depression | Commercial | ActiCal, GENEActiv | Activinsights, Philips | Smart band | Wrist | Windows | NR | PC | Docking station |
| Miranda [58] | Anxiety | Commercial | Empatica E3, Muse, Zephyr HxM | Empatica, InteraXon, Medtronic | Smart band | Chest, head, wrist | Android, iOS, Linux, Mac OS, Windows | NR | PC, server, smartphone, tablet | Bluetooth, wired |
| Mullick [59] | Depression | Commercial | Fitbit Inspire HR | Fitbit Inc | Smart band | Wrist | Android, iOS, Mac OS, Windows | PC, smartphone, tablet | Server | Bluetooth, Internet |
| Narziev [60] | Depression | Commercial | Gear S3 | Samsung | Smartwatch | Wrist | Android, iOS | Smartphone | Server | Bluetooth |
| Nath [61] | Anxiety | Non-commercial | NR | NA | Smart band | Wrist | Windows | Smartphone | Server | Bluetooth, Internet |
| Nguyen [62] | Depression | Commercial | Actiwatch AW4 | Cambridge Neurotechnology | Smartwatch | Wrist | Windows | NR | PC | Docking station |
| Nishimura [63] | Anxiety, depression | Commercial | Fitbit Charge 3 | Fitbit Inc | Smart band | Wrist | Android, iOS, Mac OS, Windows | PC, smartphone, tablet | Server | Bluetooth, Internet |
| Opoku Asare [64] | Depression | Commercial | Oura Ring | Oura | Smart ring | Finger | Android, iOS | Smartphone | Server | Bluetooth, Internet |
| Pacheco-Gonzalez [65] | Depression | Commercial | Actiwatch AW4 | Cambridge Neurotechnology | Smartwatch | Wrist | Windows | NR | PC | Docking station |
| Pedrelli [66] | Depression | Commercial | Empatica E4 | Empatica | Smart band | Wrist | Android, iOS, Mac OS, Windows | NR | PC, server, smartphone | Bluetooth, docking station |
| Qian [67] | Depression | Commercial | Ruputer | Seiko Instruments Inc | Smartwatch | Wrist | Mac OS, Windows | NR | PC | Docking station |
| Raihan [68] | Depression | Commercial | Actiwatch AW4 | Cambridge Neurotechnology | Smartwatch | Wrist | Windows | NR | PC | Docking station |
| Rodríguez-Ruiz [69] | Depression | Commercial | Actiwatch AW4 | Cambridge Neurotechnology | Smart band | Wrist | Windows | NR | PC | Docking station |
| Rodríguez-Ruiz [70] | Depression | Commercial | Actiwatch AW4 | Cambridge Neurotechnology | Smart band | Wrist | Windows | NR | PC | Docking station |
| Rodríguez-Ruiz [71] | Depression | Commercial | Actiwatch AW4 | Cambridge Neurotechnology | Smart band | Wrist | Windows | NR | PC | Docking station |
| Rother [72] | Anxiety | Non-commercial | NR | NA | Smart band | Waist | Windows | NR | PC | Wired |
| Rykov [73] | Depression | Commercial | Fitbit Charge HR, Fitbit Charge 2 | Fitbit Inc | Smart band | Wrist | Android, iOS, Mac OS, Windows | PC, smartphone, tablet | Server | Bluetooth, Internet |
| Saha [74] | Anxiety | Commercial | Vivosmart | Garmin | Smart band | Wrist | Android, iOS, Mac OS, Windows | NR | PC, server, smartphone, tablet | Bluetooth |
| Šalkevicius [75] | Anxiety | Commercial | Empatica E4 | Empatica | Smart band | Wrist | Android, iOS, Mac OS, Windows | NR | PC, server, smartphone | Bluetooth, docking station |
| Scism [76] | Anxiety, depression | Commercial | 3-Space Sensor | YEI Technology | Smart band | Head, waist | NR | NR | PC | Bluetooth, Internet, removable media, wired |
| Shah [77] | Depression | Commercial | Galaxy watch | Samsung | Smartwatch | Wrist | Android, iOS | NR | PC, smartphone, tablet | Bluetooth |
| Shaukat-Jali [78] | Anxiety | Commercial | Empatica E4 | Empatica | Smart band | Wrist | Android, iOS, Mac OS, Windows | NR | PC, server, smartphone | Bluetooth, docking station |
| Tazawa [79] | Depression | Commercial | Silmee W20 | TDK Corporation | Smart band | Wrist | Android, iOS | Smartphone, Silmee L20 gateway device | Server | Bluetooth, Internet |
| Tiwari [80] | Anxiety | Commercial | Fitbit Charge 2, Omsignal Smart-Shirt | Fitbit Inc, OMsignal Smart Clothing | Smart band, smart shirt | Chest, Wrist | Android, iOS, Mac OS, Windows | PC, smartphone, tablet | Server | Bluetooth, Internet |
| Tsai [81] | Anxiety | Commercial | Vivosmart 4 | Garmin | Smart band | Wrist | Android, iOS, Mac OS, Windows | NR | PC, server, smartphone, tablet | Bluetooth, ANT+ |
| Valenza [82] | Depression | Non-commercial | PSYCHE | Smartex | Smart shirt | Chest | NR | NR | Server | Removable media |
| Wang [83] | Depression | Commercial | Microsoft Band 2 | Microsoft | Smartwatch | Wrist | Android, iOS, Windows | Smartphone | Server | Bluetooth, Internet |
| Xu [84] | Depression | Commercial | Fitbit Flex 2 | Fitbit Inc | Smart band | Wrist | Android, iOS, Mac OS, Windows | PC, smartphone, tablet | Server | Bluetooth, Internet |
| Yadav [85] | Anxiety | Commercial | Actiwave Cardio, Empatica E4 | Empatica, Camntech | Smart band, smart adhesive electrodes | Chest, wrist | Android, iOS, Mac OS, Windows | NR | PC, server, smartphone | Bluetooth, docking station |
| Zanella-Calzada [86] | Depression | Commercial | Actiwatch AW4 | Cambridge Neurotechnology | Smartwatch | Wrist | Windows | NR | PC | Docking station |
| Zheng [87] | Anxiety | Commercial, non-commercial | MindWave Mobile | Neurosky | Smart headset, smart glasses | Eyes, head | Android, iOS, Mac OS, Windows | NR | PC, smartphone, tablet | Bluetooth |
| NR: Not reported, PC: Personal computer | | | | | | | | | | |
