# Supplementary material for: Wearable Artificial Intelligence for Anxiety and Depression: Scoping Review
Source: J Med Internet Res. 2023 Jan 19;25:e42672. doi: 10.2196/42672 (PMC9896355; doi:10.2196/42672)
Supplement: Multimedia Appendix 6 [file jmir_v25i1e42672_app6.docx]

**Multimedia Appendix 6: Features of sensors of wearable devices**

| Study [Ref] | Sensors | Sensing approach | Sensing type | Measured biosignals |
| --- | --- | --- | --- | --- |
| Adamczyk [19] | Accelerometer | Opportunistic, participatory | Passive | Activity measures, sleep measures |
| Aminifar [20] | Accelerometer | Opportunistic, participatory | Passive | Activity measures, sleep measures |
| Arsalan [21] | EEG sensor | Opportunistic | Passive | EEG |
| Arsalan [22] | EEG sensor | Opportunistic | Passive | EEG |
| Bai [23] | Accelerometer, PPG sensor | Opportunistic | Active, Passive | Activity measures, heart rate measures, sleep measures |
| Bennett [24] | Accelerometer | Opportunistic | Passive | Activity measures, sleep measures |
| Chikersal [25] | Accelerometer | Opportunistic | Passive | Activity measures, sleep measures |
| Cho [26] | Accelerometer, altimeter, PPG sensor | Opportunistic | Active, Passive | Activity measures, heart rate measures, sleep measures |
| Choi [27] | Accelerometer, light sensor | Opportunistic | Passive | Activity measures, light exposure |
| Choi [28] | Accelerometer, EDA sensor, PPG sensor, thermometer | Opportunistic, participatory | Active, Passive | Activity measures, EDA, heart rate measures, skin temperature, sleep measures |
| Coutts [29] | Accelerometer, PPG sensor | Opportunistic | Active, Passive | Heart rate measures |
| Dai [30] | Accelerometer, PPG sensor | Opportunistic | Active, Passive | Activity measures, heart rate measures, sleep measures |
| Feng [31] | Accelerometer, altimeter, PPG sensor | Opportunistic | Active, Passive | Activity measures, heart rate measures, sleep measures |
| Frogner [32] | Accelerometer | Opportunistic, participatory | Passive | Activity measures, sleep measures |
| Fukuda [33] | Accelerometer, altimeter, PPG sensor | Opportunistic | Active, Passive | Activity measures, heart rate measures, sleep measures |
| Galvan-Tejada [34] | Accelerometer | Opportunistic, participatory | Passive | Activity measures, sleep measures |
| Garcia-Ceja [35] | Accelerometer | Opportunistic, participatory | Passive | Activity measures, sleep measures |
| Garcia-Ceja [36] | Accelerometer | Opportunistic, participatory | Passive | Activity measures, sleep measures |
| Ghandeharioun [37] | Accelerometer, EDA sensor, PPG sensor, thermometer | Opportunistic, participatory | Active, Passive | Activity measures, EDA, heart rate measures, skin temperature, sleep measures |
| Griffiths [38] | Accelerometer, altimeter, PPG sensor | Opportunistic | Active, Passive | Activity measures, heart rate measures, sleep measures |
| Gu [39] | Accelerometer, humidity sensor, gyroscope, microphone, thermometer | Opportunistic | Passive | Activity measures, audio, skin humidity, skin temperature |
| Ihmig [40] | ECG sensor, EDA sensor, piezoelectric sensor, PPG sensor | Opportunistic | Active, Passive | ECG, EDA, heart rate measures, respiratory measures |
| Jacobson [41] | Accelerometer | Opportunistic, participatory | Passive | Activity measures, sleep measures |
| Jacobson [42] | Accelerometer, light sensor | Opportunistic | Passive | Activity measures, light exposure, sleep measures |
| Jakobsen [43] | Accelerometer | Opportunistic, participatory | Passive | Activity measures, sleep measures |
| Jin [44] | Accelerometer, humidity sensor, gyroscope, microphone, thermometer | Opportunistic | Passive | Activity measures, audio, skin humidity, skin temperature |
| Khan [45] | Accelerometer, gyroscope | Opportunistic | Passive | Activity measures |
| Kim [46] | Accelerometer, light sensor | Opportunistic, participatory | Passive | Activity measures, light exposure, mood status, sleep measures |
| Kulam [47] | Accelerometer | Opportunistic, participatory | Passive | Activity measures, sleep measures |
| Kumar [48] | Accelerometer | Opportunistic, participatory | Passive | Activity measures, sleep measures |
| Llamocca [49] | Accelerometer, light sensor | Opportunistic | Passive | Activity measures, light exposure, sleep measures |
| Lu [50] | Accelerometer, altimeter, PPG sensor | Opportunistic | Active, Passive | Activity measures, heart rate measures, sleep measures |
| Mahendran [51] | Accelerometer, PPG sensor | Opportunistic | Active, Passive | Activity measures, heart rate measures, sleep measures |
| Makhmutova [52] | Accelerometer | Opportunistic | Passive | Activity measures, sleep measures |
| Mallikarjun [53] | EEG sensor | Opportunistic | Passive | EEG |
| McGinnis [54] | Accelerometer, compass, gyroscope, microphone | Opportunistic | Passive | Activity measures, audio |
| McGinnis [55] | Accelerometer, compass, gyroscope | Opportunistic | Passive | Activity measures |
| McGinnis [56] | Accelerometer, compass, gyroscope | Opportunistic | Passive | Activity measures |
| Minaeva [57] | Accelerometer, light sensor | Opportunistic, participatory | Passive | Activity measures, light exposure, sleep measures |
| Miranda [58] | Accelerometer, ECG sensor, EDA sensor, PPG sensor, thermometer | Opportunistic, participatory | Active, Passive | Activity measures, EDA, EEG, heart rate measures, skin temperature, sleep measures |
| Mullick [59] | Accelerometer, PPG sensor | Opportunistic | Active, Passive | Activity measures, heart rate measures, sleep measures |
| Narziev [60] | Accelerometer, barometer, gyroscope, PPG sensor | Opportunistic | Active, Passive | Activity measures, air pressure, heart rate measures, sleep measures |
| Nath [61] | EDA sensor, PPG sensor | Opportunistic | Active, Passive | EDA data, heart rate measures |
| Nguyen [62] | Accelerometer | Opportunistic, participatory | Passive | Activity measures, sleep measures |
| Nishimura [63] | Accelerometer, altimeter, PPG sensor | Opportunistic | Active, Passive | Activity measures, heart rate measures, sleep measures |
| Opoku Asare [64] | Accelerometer, gyroscope, PPG sensor, thermometer | Opportunistic | Active, Passive | Activity measures, heart rate measures, respiratory measures, skin temperature, sleep measures, ultraviolet level |
| Pacheco-Gonzalez [65] | Accelerometer | Opportunistic, participatory | Passive | Activity measures, sleep measures |
| Pedrelli [66] | Accelerometer, EDA sensor, PPG sensor, thermometer | Opportunistic, participatory | Active, Passive | Activity measures, EDA, heart rate measures, skin temperature, sleep measures |
| Qian [67] | Accelerometer | Opportunistic | Passive | Activity measures, sleep measures |
| Raihan [68] | Accelerometer | Opportunistic, participatory | Passive | Activity measures, sleep measures |
| Rodríguez-Ruiz [69] | Accelerometer | Opportunistic, participatory | Passive | Activity measures, sleep measures |
| Rodríguez-Ruiz [70] | Accelerometer | Opportunistic, participatory | Passive | Activity measures, sleep measures |
| Rodríguez-Ruiz [71] | Accelerometer | Opportunistic, participatory | Passive | Activity measures, sleep measures |
| Rother [72] | Accelerometer, gyroscope, PPG sensor, EDA sensor | Opportunistic | Active, Passive | Activity measures, EDA, heart rate measures, respiratory rate measures |
| Rykov [73] | Accelerometer, altimeter, PPG sensor | Opportunistic | Active, Passive | Activity measures, heart rate measures, sleep measures |
| Saha [74] | Accelerometer, altimeter, PPG sensor | Opportunistic | Active, Passive | Activity measures, heart rate measures, sleep measures |
| Šalkevicius [75] | Accelerometer, EDA sensor, PPG sensor, thermometer | Opportunistic | Active, Passive | Activity measures, EDA, heart rate measures, skin temperature, sleep measures |
| Scism [76] | Accelerometer, compass, gyroscope | Opportunistic | Passive | Activity measures |
| Shah [77] | Accelerometer, compass, gyroscope, PPG sensor, light sensor | Opportunistic | Active, Passive | Activity measures, heart rate measures, light exposure, sleep measures |
| Shaukat-Jali [78] | Accelerometer, EDA sensor, PPG sensor, thermometer | Opportunistic | Active, Passive | Activity measures, EDA, heart rate measures, skin temperature, sleep measures |
| Tazawa [79] | Accelerometer, PPG sensor, thermometer, UV sensor | Opportunistic | Active, Passive | Activity measures, heart rate measures, skin temperature, sleep measures, ultraviolet level |
| Tiwari [80] | Accelerometer, altimeter, ECG sensor, PPG sensor | Opportunistic | Active, Passive | Activity measures, ECG data, heart rate measures, respiratory measures, sleep measures |
| Tsai [81] | Accelerometer, altimeter, oximeter, PPG sensor | Opportunistic | Active, Passive | Activity measures, blood oxygen saturation, heart rate measures, sleep measures |
| Valenza [82] | Accelerometer, ECG sensor, piezoresistive sensor | Opportunistic | Passive | Activity measures, heart rate measures, respiratory measures, sleep measures |
| Wang [83] | Accelerometer, barometer, EDA sensor, GPS, gyroscope, light sensor, microphone, PPG sensor, thermometer, UV sensor | Opportunistic | Active, Passive | Activity measures, air pressure, audio, EDA, heart rate measures, light exposure, location, skin temperature, sleep measures, ultraviolet level |
| Xu [84] | Accelerometer | Opportunistic | Passive | Activity measures, sleep measures |
| Yadav [85] | Accelerometer, ECG sensor, EDA sensor, PPG sensor, thermometer | Opportunistic | Active, Passive | Activity measures, ECG, EDA, heart rate measures, sleep measures, skin temperature |
| Zanella-Calzada [86] | Accelerometer | Opportunistic, participatory | Passive | Activity measures, sleep measures |
| Zheng [87] | EEG sensor, PPG sensor | Opportunistic | Active, Passive | EEG, heart rate measures |
| ECG: Electrocardiogram, EDA: Electrodermal activity, EEG: Electroencephalogram, PPG: photoplethysmogram, UV: Ultraviolet | | | | |
