# Supplementary material for: Wearable Artificial Intelligence for Anxiety and Depression: Scoping Review
Source: J Med Internet Res. 2023 Jan 19;25:e42672. doi: 10.2196/42672 (PMC9896355; doi:10.2196/42672)
Supplement: Multimedia Appendix 7 [file jmir_v25i1e42672_app7.docx]

**Multimedia Appendix 7: Features of AI algorithms**

| Study [Ref] | AI category | Problem solving approach | AI algorithm | Aim of AI algorithm | Ground truth assessment | Validation approach | Performance measures |
| --- | --- | --- | --- | --- | --- | --- | --- |
| Adamczyk [19] | ML | Classification | LogR, RF, SVM | Diagnosis/ screening | MADRS | Nested | AUC, BACC, F1, MCC, PREC, ROC, SENS, SPEC |
| Aminifar [20] | ML | Classification | DT, ERT, ID3, RF, SVM, XGBoost | Monitoring | MADRS | LOOCV | ACC, F1, MCC |
| Arsalan [21] | ML, DL | Classification | LogR, MLP, RF | Diagnosis/ screening | STAI | K-fold | ACC, F1, Kappa, MAE, PREC, RAE, RMSE, RRSE, SENS |
| Arsalan [22] | ML, DL | Classification | LogR, MLP, RF | Diagnosis/ screening | STAI | K-fold | ACC, F1, Kappa, MAE, RMSE, SENS, SPEC |
| Bai [23] | ML | Classification | DT, LogR, NB, KNN, RF, SVM | Monitoring | PHQ-9 | K-fold | ACC, SENS |
| Bennett [24] | DL | Classification | MLP | Prediction | PHQ-9 | K-fold | ACC |
| Chikersal [25] | ML | Classification | AdaBoost, GB, LogR | Prediction | BDI-II | LOOCV | ACC, F1 |
| Cho [26] | ML | Classification | RF | Prediction | DSM-5 | Hold-out | ACC, AUC, SENS, SPEC |
| Choi [27] | ML, DL | Classification | LogR, MLP, SVC, XGBoost | Diagnosis/ screening | PHQ-9 | Hold-out | ACC, AUC, F1, PREC, ROC, SENS |
| Choi [28] | ML, DL | Classification | DT, GB, KNN, MLP, RF, SVM, XGBoost | Monitoring | GDS, PHQ-9 | K-fold | ACC, F1, SENS |
| Coutts [29] | DL | Classification | LSTM | Prediction | DASS, STAI | Hold-out | ACC |
| Dai [30] | ML, DL | Classification | AdaBoost, ANN, LogR, GBDT, New proposed model (MTL), RF, SVM | Prediction | PHQ-9 | K-fold | AUC, AUPRC |
| Feng [31] | ML | Classification, clustering | GMM, SVM | Prediction | STAI | Hold-out | ACC |
| Frogner [32] | DL | Classification | CNN | Diagnosis/ screening | MADRS | Hold-out, K-fold, LOOCV | ACC, F1, MSE, PREC, SENS, SPEC |
| Fukuda [33] | ML | Classification | RF | Prediction | DAMS | LOOCV | ACC, F1, PREC, SENS |
| Galvan-Tejada [34] | ML | Classification | RF | Monitoring | MADRS | Hold-out, K-fold | AUC, SENS, SPEC |
| Garcia-Ceja [35] | ML, DL | Classification | AdaBoost, ANN, DT, KNN, GP, NB, QDA, RF, SVM, ZeroR | Diagnosis/ screening | MADRS | K-fold | ACC, F1, MCC, PREC, SENS, SPEC |
| Garcia-Ceja [36] | ML, DL | Classification | DNN, RF | Diagnosis/ screening | MADRS | LOOCV | ACC, F1, MCC, PREC, SENS, SPEC |
| Ghandeharioun [37] | ML | Regression | AdaBoost, elasticNet, huber methods, Gaussian boosting, GP, LASSO, LinR, RANSAC, RF, RR, Theil-Sen | Monitoring | HDRS | Hold-out, K-fold, LOOCV | RMSE |
| Griffiths [38] | ML, DL | Classification | DT, KNN, LogR, NB, RF, SVM | Diagnosis/ screening | PHQ-9 | K-fold | ACC, F1, PREC, SENS |
| Gu [39] | ML | Classification | K-means | Diagnosis/ screening | STAI | NR | ACC |
| Ihmig [40] | ML | Classification | DT, Ensemble model, KNN, LDA, NB, QDA, SVM | Diagnosis/ screening | Observation, self-rated question | K-fold | ACC, AUC, Kappa, THR, TLR, TMR |
| Jacobson [41] | ML | Classification, regression | XGBoost | Monitoring | MADRS | LOOCV | ACC, Kappa, r, SENS, SPEC |
| Jacobson [42] | ML, DL | Classification | ANN, GLM, GP, KNN, RF, RR, SR, SVM, XGBoost | Prediction | CIDI | K-fold | AUC, BACC, SENS, SPEC |
| Jakobsen [43] | ML, DL | Classification | CNN, DNN, RF | Diagnosis/ screening | MADRS | LOOCV | ACC, MCC, SPEC, SENS |
| Jin [44] | DL | Classification | CNN, LSTM | Monitoring | BDI-II, STAI | Hold-out | F1 |
| Khan [45] | ML, DL | Classification | CNN, DNN, GB, LSTM, RF | Monitoring | Clinician assessment | Hold-out, K-fold, LOOCV | ACC |
| Kim [46] | ML | Classification | BT, DT, LogR, RF | Diagnosis/ screening | HDRS, GDS | Hold-out | ACC, AUC, F1, PREC, SENS, SPEC |
| Kulam [47] | DL | Classification | CNN, LSTM | Diagnosis/ screening | MADRS | K-fold | ACC, F1, MCC, PREC, SENS, SPEC |
| Kumar [48] | ML, DL | Classification | ANFIS, CNN | Diagnosis/ screening | MADRS | Hold-out | ACC, MAE |
| Llamocca [49] | ML | Classification | DT, LogR, RF, SVM | Diagnosis/ screening | Clinician assessment | Hold-out | ACC |
| Lu [50] | ML | Classification, regression | LASSO, RR | Monitoring | DSM-5, QIDS | LOOCV | F1, R-Squared |
| Mahendran [51] | ML | Classification | Ensemble model, LogR, RF | Diagnosis/ screening | HDRS | Hold-out, K-fold | ACC, AUC, F1, PREC, SENS, SPEC |
| Makhmutova [52] | ML | Classification | DART boosting, LightGBM | Prediction | PHQ-9 | K-fold | AACC, AUPRC, BACC, F1, Kappa, SENS, SPEC |
| Mallikarjun [53] | ML | Classification | CN2 Rule Inducer, NB, RF, Tree, SVM | Diagnosis/ screening | PHQ-9 | LOOCV | ACC, FPR, PREC, SENS, SPEC |
| McGinnis [54] | ML | Classification | DT | Diagnosis/ screening | CBCL, K-SADS | LOOCV | ACC, AUC, SENS, SPEC |
| McGinnis [55] | ML | Classification | DT, LogR, KNN, SVM | Diagnosis/ screening | DSM-IV | LOOCV | ACC, AUC, ROC |
| McGinnis [56] | ML | Classification | DT, LogR, KNN, SVM | Diagnosis/ screening | DSM-IV | LOOCV | ACC, AUC, SENS, SPEC |
| Minaeva [57] | ML | Classification | LogR | Diagnosis/ screening | CIDI | External validation | AUC, Nagelkerke R-Squared, SENS, SPEC, Youden index |
| Miranda [58] | ML | Classification | SVM | Monitoring | Observation | LOOCV | PREC, SENS |
| Mullick [59] | ML | Regression | AdaBoost, elasticNet, extra trees, GB, LASSO, RF, XGBoost | Prediction | PHQ-9 | LOOCV | MAE, MAPE, MSE, RMSE |
| Narziev [60] | ML | Classification | RF, SVM | Monitoring | BDI‐II, DSM‐5, PHQ-9 | Hold-out, K-fold | ACC, F1, PREC, SENS |
| Nath [61] | ML | Classification | LogR, RF, SVM | Diagnosis/ screening | STAI | Hold-out | ACC, F1 |
| Nguyen [62] | DL | Classification | CNN | Diagnosis/ screening | MADRS | Hold-out, K-fold | ACC, AUC, F1, MCC, PREC, SENS, SPEC |
| Nishimura [63] | ML | Classification | LightGBM | Diagnosis/ screening | DAMS | K-fold | ACC, F1 |
| Opoku Asare [64] | ML | Classification | KNN, LogR, RF, SVM, XGBoost | Monitoring | DASS | Nested, time-series | ACC, AUC, F1, PREC, SENS |
| Pacheco-Gonzalez [65] | ML | Classification | RF | Diagnosis/ screening | MADRS | NR | AUC, ROC, SENS, SPEC |
| Pedrelli [66] | ML | Regression | Ensemble model, RF | Monitoring | HDRS | Hold-out, K-fold | MAE, r, RMSE |
| Qian [67] | ML | Classification | SVM | Diagnosis/ screening | NR | Hold-out | ACC, AUC, BACC, F1, PREC, SENS, SPEC |
| Raihan [68] | ML, DL | Classification | AdaBoost, ANN, RF | Diagnosis/ screening | MADRS | K-fold | ACC, F1, Kappa, MCC, PREC, SENS |
| Rodríguez-Ruiz [69] | ML | Classification | RF | Diagnosis/ screening | MADRS | Hold-out | ACC, AUC, SENS, SPEC |
| Rodríguez-Ruiz [70] | ML | Classification | RF | Diagnosis/ screening | MADRS | Hold-out | ACC, NPV, PREC, SENS, SPEC |
| Rodríguez-Ruiz [71] | ML | Classification | RF | Diagnosis/ screening | BPRS, MADRS | K-fold | ACC, AUC, F1, MCC, PREC, SENS |
| Rother [72] | ML, DL | Classification | DT, MLP, SVM | Monitoring | NR | NR | F1, PREC, SENS |
| Rykov [73] | ML | Classification, regression | XGBoost | Diagnosis/ screening | PHQ-9 | K-fold | ACC, AUC, Kappa, MAE, NPV, PCC, PREC, R-Squared, RMSE, SENS, SPEC |
| Saha [74] | ML, DL | Clustering, regression | elasticNet, HC, GBR, K-means, MLP, PAM, RR, SVR, XGBoost | Diagnosis/ screening | STAI | Hold-out, K-fold | Connectivity, Dunn index, r, Silhouette score, SMAPE |
| Šalkevicius [75] | ML | Classification | SVM | Diagnosis/ screening | SUDS | K-fold, LOOCV | ACC |
| Scism [76] | ML | Classification | DT | Diagnosis/ screening | CBCL, K-SADS | LOOCV | ACC |
| Shah [77] | ML | Regression | AdaBoost, elasticNet, GB, PR, RF, SVM, VR | Prediction | PHQ-9 | Nested | MAE, MAPE |
| Shaukat-Jali [78] | ML | Classification | DT, KNN, RF, SVM | Diagnosis/ screening | LSAS, SPSQ | K-fold | ACC |
| Tazawa [79] | ML | Classification, regression | RF, SVM, XGBoost | Diagnosis/ screening | HDRS | K-fold | ACC, MAE, r, SENS, SPEC |
| Tiwari [80] | ML | Classification | SVM | Diagnosis/ screening | NR | K-fold | BACC, F1, SENS, SPEC |
| Tsai [81] | ML | Classification | Adaboost, DT, LDA, RF, RGF, XGBoost | Prediction | PDSS | Hold-out, K-fold | ACC, AUC, F1, ROC, SENS, SPEC |
| Valenza [82] | DL | Classification | MLP | Diagnosis/ screening | DSM-IV, QIDS, YMRS | Hold-out, K-fold | ACC |
| Wang [83] | ML | Regression | LASSO | Diagnosis/ screening | PHQ-4, PHQ-8 | K-fold | MAE, r |
| Xu [84] | ML | Classification | AdaBoost | Prediction | BDI-II | Hold-out, LOOCV | ACC, F1, PREC, SENS |
| Yadav [85] | ML, DL | Classification, clustering, regression | FNN, K-means, LinR | Diagnosis/ screening | CAI | LOOCV | SRCC |
| Zanella-Calzada [86] | ML | Classification | RF | Monitoring | MADRS | Hold-out | ACC, AUC, BACC, NPV, ROC, PREC, SENS, SPEC |
| Zheng [87] | ML | Classification | KNN, SVM | Diagnosis/ screening | CSAI-2 | K-fold, repeated random subsampling | ACC |
| AACC: Adjacent Accuracy, ACC: Accuracy, ANFIS: Adaptive neuro fuzzy inference system, AUC: Area Under the Curve, AUPRC: Area Under the Precision Recall Curve, BACC: Balanced Accuracy, BDI-II: Beck Depression Inventory-II, BDP-GMM: Bayesian Dirichlet process Gaussian mixture model, BPRS: Brief Psychiatric Rating Scale, BT: Boosted Trees, CAI: Communication Anxiety Inventory, CBCL: Child Behavior Checklist, CIDI: Composite international diagnostic interview, CNN: Convolutional Neural Network, CSAI: Competitive State Anxiety Inventory, DAMS: Depression and Anxiety Mood Scale, DART: Dropouts Meet Multiple Additive Regression Trees, DASS: Depression Anxiety Stress Scales, DBSCAN: Density-based spatial clustering of applications with noise, DeSGE: Deep Stacked Generalization Ensemble Learning, DL: Deep learning, DNN: Deep Neural Network, DSM: Diagnostic and Statistical Manual of Mental Health, DT: Decision tree, ERT: Extremely Randomized Trees, F1: F score, FNN: Feed-Forward Neural Network, FPR: False Positive Rate, GB: Gradient Boosting, GBDT: Gradient Boosting Decision Trees, GBR: Gradient Boosted Regression, GDS: Geriatric Depression Scale, GLM: General Linear Model, GMM: Gaussian mixture models, GP: Gaussian Process, HC: Hierarchical clustering, HDRS: Hamilton Depression Rating Scale, ID3: Iterative Dichotomiser 3, KNN: K-Nearest Neighbors, K-SADS: Kiddie-Schedule for Affective Disorders and Schizophrenia for School-Aged Children, LASSO: Least Absolute Shrinkage and Selection Operator, LDA: Linear discriminant analysis, LightGBM: Light Gradient Boosting Machine, LinR: Linear regression, LogR: Logistic regression, LSAS: Liebowitz Social Anxiety Scale, MADRS: Montgomery-Asberg Depression Rating Scale, MAE: Mean Absolute Error, MAPE: Mean Absolute Percentage Error, MCC: Matthews correlation coefficient, ML: Machine learning, MLM: Multi Level Modeling, MSE: Mean Squared Error, NA: Not applicable, NB: Naive Bayes, NN: Neural Network, NPV: Negative Predictive Value, NR: Not reported, OneR: One Rule, PCC: Pearson correlation coefficient, PCC: Pearson correlation coefficient, PDSS: Panic Disorder Severity Scale, PHQ-9: Patient Health Questionnaire-9, PPD-ERT: Privacy preserving distributed extremely randomized trees, PR: Poisson regression, PRES: Precision, QDA: Quadratic Discriminant Analysis, QIDS: Quick Inventory of Depressive Symptomatology, r: correlation coefficient, RAE: Relative Absolute Error, RBF: Radial Basis Function, RF: Random Forest, RGF: regularized greedy forests, RMSE: Root Mean Square Error, ROC: Receiver Operating Characteristic Curve, RR: Ridge Regression, RRSE: Root Relative Square Error, R-Squared: Coefficient of determination, SENS: Sensitivity, SMAPE: Symmetric Mean Absolute Percentage Error, SPES: Specificity, SPSQ: Social Phobia Screening Questionnaire, SR: Splines Regression, SRCC: Spearman's Rank Correlation Coefficient, STAI: State-Trait Anxiety Inventory, SUDS: Subjective units of distress scale, SVM: Support Vector Machine, THR: True High Rate, TLR: True Low Rate, TMR: True Medium Rate, VR: Voting regressor, XGBoost: extreme gradient boosting, YMRS: Young Mania Rating Scale | | | | | | | |
