# Supplementary material for: Wearable Artificial Intelligence for Anxiety and Depression: Scoping Review
Source: J Med Internet Res. 2023 Jan 19;25:e42672. doi: 10.2196/42672 (PMC9896355; doi:10.2196/42672)
Supplement: Multimedia Appendix 8 [file jmir_v25i1e42672_app8.docx]

**Multimedia Appendix 8: Features of data used in AI algorithms**

| Study [Ref] | Dataset size | Data source | Data types | Data input to AI algorithm | Number of features |
| --- | --- | --- | --- | --- | --- |
| Adamczyk [19] | NR | Open | WD-based, self-reported | Activity data | 5, 10 |
| Aminifar [20] | NR | Open | WD-based | Activity data | NR |
| Arsalan [21] | 168 | Closed | WD-based | EEG data | 6 |
| Arsalan [22] | NR | Closed | WD-based | EEG data | 5 |
| Bai [23] | 950 | Closed | WD-based, non-WD based | Activity data, heart rate data, location, sleep data, smartphone usage data, social interaction | 4, 5, 7-13, 17, 18, 20, 34-37, 44, 48, 75 |
| Bennett [24] | NR | Closed | WD-based, non-WD based, self-reported data | Activity data, loneliness level, quality of life, sleep data, social interaction | 6 |
| Chikersal [25] | NR | Closed | WD-based, non-WD based | Activity data, location, sleep data, smartphone usage data, social interaction | 7 |
| Cho [26] | NR | Closed | WD-based, self-reported | Activity data, heart rate data, light exposure, mood status, sleep data | 13 |
| Choi [27] | 1570144 | Open | WD-based, self-reported | Circadian rhythms | 15 |
| Choi [28] | NR | Closed | WD-based | Activity data, EDA data, heart rate data, skin temperature | 4, 156, 160 |
| Coutts [29] | NR | Closed | WD-based | Heart rate data | NR |
| Dai [30] | NR | Closed | WD-based, self-reported | Activity data, coping, demographic data, depression level, heart rate data, negative problem orientation, post-traumatic stress disorder status, psychiatric status, sleep data | 20 |
| Feng [31] | NR | Open | WD-based | Activity data, heart rate data | NR |
| Frogner [32] | NR | Open | WD-based | Activity data | NR |
| Fukuda [33] | NR | Closed | WD-based | Sleep data | 13 |
| Galvan-Tejada [34] | NR | Open | WD-based | Activity data | 2, 5, 38 |
| Garcia-Ceja [35] | NR | Closed | WD-based | Activity data | 3 |
| Garcia-Ceja [36] | NR | Open | WD-based | Activity data | 3 |
| Ghandeharioun [37] | 1707 | Closed | WD-based, non-WD based, self-reported data | Activity data, alcohol, drug, and caffeine consumption, anxiety level, EDA data, location, mood status, sleep data, smartphone usage data, social interaction, stress level | 8 |
| Griffiths [38] | NR | Closed | WD-based | Activity data, sleep data | 6, 10, 36 |
| Gu [39] | NR | Closed | WD-based | Audio data | 2 |
| Ihmig [40] | NR | Closed | WD-based, non-WD based | EDA data, heart rate data | 6 |
| Jacobson [41] | NR | Open | WD-based | Activity data | NR |
| Jacobson [42] | 13250 | Closed | WD-based | Activity data, sleep data | 50 |
| Jakobsen [43] | NR | Open | WD-based | Activity data | 3 |
| Jin [44] | 3600 | Closed | WD-based | Activity data, audio data | 30 |
| Khan [45] | 709583 | Closed | WD-based | Activity data | 11 |
| Kim [46] | NR | Closed | WD-based | Activity data, depression level, light exposure, sleep data | 4 |
| Kulam [47] | NR | Open | WD-based | Activity data | 4 |
| Kumar [48] | NR | Open | WD-based | Activity data | 14 |
| Llamocca [49] | NR | Closed | WD-based, self-reported | Activity data, irritability level, motivation level, sleep data | NR |
| Lu [50] | NR | Closed | WD-based, non-WD based | Activity data, depression level, heart rate data, location, sleep data | 8, 36 |
| Mahendran [51] | 7200 | Closed | WD-based, self-reported | Activity data, heart rate data, sleep data | 16 |
| Makhmutova [52] | NR | Closed | WD-based, self-reported | Current therapies, demographic data, depression level, health care utilization, lifestyle changes, medical history, sleep data | 13, 30 |
| Mallikarjun [53] | NR | Closed | WD-based | EEG data | 17 |
| McGinnis [54] | NR | Closed | WD-based | Activity data, audio data | 10 |
| McGinnis [55] | NR | Closed | WD-based | Activity data | 10 |
| McGinnis [56] | NR | Closed | WD-based | Activity data | 10 |
| Minaeva [57] | NR | Closed | WD-based, self-reported | Activity data, behavioural data, circadian rhythms, demographic data, emotional data, sleep data | 2, 3, 11 |
| Miranda [58] | 424 | Closed | WD-based | EDA data, heart rate data | 9 |
| Mullick [59] | NR | Closed | WD-based, non-WD based | Activity data, heart rate data, location, sleep data, smartphone usage data, social interaction | 61 |
| Narziev [60] | 2046 | Closed | WD-based, non-WD based, self-reported data | Activity data, light exposure, food intake, heart rate data, mood status, sleep data, smartphone use data, social interaction | 22 |
| Nath [61] | 4853 | Closed | WD-based | EDA data, heart rate data | 10, 11, 14, 15 |
| Nguyen [62] | 814 | Open | WD-based | Activity data | NR |
| Nishimura [63] | NR | Closed | WD-based, non-WD based, self-reported data | Activity data, behavioural data, heart rate data, sleep data, weather data | 22 |
| Opoku Asare [64] | NR | Closed | WD-based, non-WD based, self-reported data | Activity data, heart rate data, location, mood status, sleep data, smartphone usage data | 45 |
| Pacheco-Gonzalez [65] | NR | Open | WD-based | Activity data | NR |
| Pedrelli [66] | NR | Closed | WD-based, non-WD based | Activity data, EDA data, heart rate data, location, sleep data, smartphone usage data, social interaction, weather data | 39 |
| Qian [67] | NR | Closed | WD-based | Activity data | 9 |
| Raihan [68] | NR | Open | WD-based, self-reported | Activity data, demographic data | 12 |
| Rodríguez-Ruiz [69] | 11945 | Open | WD-based | Activity data | 8 |
| Rodríguez-Ruiz [70] | NR | Open | WD-based | Activity data | 9 |
| Rodríguez-Ruiz [71] | NR | Open | WD-based | Activity data | 5, 6, 7, 8 |
| Rother [72] | NR | Closed | WD-based, non-WD based | Activity data, EDA data, heart rate data, respiratory rate measures, social interaction | 3, 4, 11 |
| Rykov [73] | NR | Closed | WD-based | Activity data, circadian rhythm, sleep data | 36 |
| Saha [74] | NR | Open | WD-based, non-WD based | Activity data, behavioural data, location, sleep data, smartphone usage data, social media data | 5173 |
| Šalkevicius [75] | NR | Closed | WD-based | EDA data, heart rate data, skin temperature | 10 |
| Scism [76] | NR | Closed | WD-based | Activity data | 13 |
| Shah [77] | NR | Closed | WD-based, non-WD based, self-reported data | Activity data, anxiety level, depression level, dietary data, heart rate data, sleep data, stress level | 43 |
| Shaukat-Jali [78] | NR | Closed | WD-based | EDA data, heart rate data, skin temperature | 3 |
| Tazawa [79] | NR | Closed | WD-based | Activity data, heart rate data, skin temperature, sleep data, UV light exposure | 63 |
| Tiwari [80] | NR | Closed | WD-based | Heart rate data | 50 |
| Tsai [81] | NR | Closed | WD-based, self-reported | Activity data, anxiety level, depression level, environmental data, heart rate data, sleep data | 61 |
| Valenza [82] | NR | Closed | WD-based | ECG data | 7 |
| Wang [83] | NR | Closed | WD-based, non-WD based, self-reported data | Activity data, heart rate data, location, sleep data, smartphone usage data, social interaction | 5, 10 |
| Xu [84] | NR | Closed | WD-based, non-WD based | Activity data, location, sleep data, smartphone usage data, social interaction | 181, 274, 455 |
| Yadav [85] | NR | Closed | WD-based, self-reported | Activity data, anxiety level, audio data, demographic data, ECG data, EDA data, heart rate data, level of preparation and knowledge on the presentation, personality metrics, skin temperature | 27 |
| Zanella-Calzada [86] | NR | Open | WD-based, self-reported | Activity data | 14 |
| Zheng [87] | NR | Closed | WD-based | EEG data, heart rate data | 9 |
| ECG: Electrocardiogram, EDA: Electrodermal activity, EEG: Electroencephalogram, NR: Not reported, UV: Ultraviolet, WD: wearable devices | | | | | |
